# A New Vision for Therapeutic Hypothermia in the Era of Targeted Temperature Management: A Speculative Synthesis

**DOI:** 10.1089/ther.2019.0001

**Published:** 2019-03-06

**Authors:** Travis C. Jackson, Patrick M. Kochanek

**Affiliations:** ^1^ John G. Rangos Research Center, UPMC Children's Hospital of Pittsburgh, Safar Center for Resuscitation Research, University of Pittsburgh, School of Medicine, Pittsburgh, Pennsylvania.; ^2^ Department of Critical Care Medicine, University of Pittsburgh, School of Medicine, Pittsburgh, Pennsylvania.

**Keywords:** therapeutic hypothermia, targeted temperature management, FGF21, RBM3, hypoxic/ischemic encephalopathy, space

## Abstract

Three decades of animal studies have reproducibly shown that hypothermia is profoundly cerebroprotective during or after a central nervous system (CNS) insult. The success of hypothermia in preclinical acute brain injury has not only fostered continued interest in research on the classic secondary injury mechanisms that are prevented or blunted by hypothermia but has also sparked a surge of new interest in elucidating beneficial signaling molecules that are increased by cooling. Ironically, while research into cold-induced neuroprotection is enjoying newfound interest in chronic neurodegenerative disease, conversely, the scope of the utility of therapeutic hypothermia (TH) across the field of acute brain injury is somewhat controversial and remains to be fully defined. This has led to the era of Targeted Temperature Management, which emphasizes a wider range of temperatures (33–36°C) showing benefit in acute brain injury. In this comprehensive review, we focus on our current understandings of the novel neuroprotective mechanisms activated by TH, and discuss the critical importance of developmental age germane to its clinical efficacy. We review emerging data on four cold stress hormones and three cold shock proteins that have generated new interest in hypothermia in the field of CNS injury, to create a framework for new frontiers in TH research. We make the case that further elucidation of novel cold responsive pathways might lead to major breakthroughs in the treatment of acute brain injury, chronic neurological diseases, and have broad potential implications for medicines of the distant future, including scenarios such as the prevention of adverse effects of long-duration spaceflight, among others. Finally, we introduce several new phrases that readily summarize the essence of the major concepts outlined by this review—namely, Ultramild Hypothermia, the “Responsivity of Cold Stress Pathways,” and “Hypothermia in a Syringe.”

## Introduction

The Egyptians recognized the medical utility of hypothermia 5000 years ago, local head cooling for traumatic brain injury (TBI) was used by Phelps in the late 1800s, and total body cooling for the treatment of head injury was first applied in 1938 by the neurosurgeon Temple Fay (Phelps, 1897; Wang *et al.*, 2006; Karnatovskaia *et al.*, 2014). In contemporary practice, therapeutic hypothermia (TH) is highly neuroprotective when applied at 33.5°C for 72 hours in selected term newborns with hypoxic/ischemic encephalopathy (HIE), where it is standard of care (Martinello *et al.*, 2017). Moreover, current guidelines recommend a targeted temperature management (TTM) of 32–36°C for 24–48 hours in the treatment of neurological injury in comatose adults with out-of-hospital cardiac arrest (Callaway *et al.*, 2015). Also, prophylactic moderate-to-deep TH is standard of care in surgeries that impair cerebral blood flow (CBF) such as for brain aneurysms or aortic arch repair (Hanel and Spetzler, 2008; Tian *et al.*, 2013). Profound hypothermia is also in clinical trials (NCT01042015) for emergency perseveration and resuscitation (EPR) of cardiac arrest from trauma (Wu *et al.*, 2006; Kutcher *et al.*, 2016). However, despite widespread use of cooling in neurocritical care, recent randomized controlled trials (RCTs) suggest that fever prevention (therapeutic normothermia or TTM in the normothermic range), or rigorous control of patient temperature at 36°C, may be equally effective versus mild TH to ∼33°C on long-term neurological outcomes in adults/children with brain injury (Adelson *et al.*, 2013; Nielsen *et al.*, 2013; Maekawa *et al.*, 2015; Moler *et al.*, 2015, 2017; Cooper *et al.*, 2018). Thus, mounting evidence suggests that the adult human brain is less protected by hypothermia compared with rodents (van der Worp *et al.*, 2007). Many factors may explain the discrepancy in preclinical data versus human trials on TH and include important age-related differences that confer greater protection in the immature brain, time to initiate cooling, which can be achieved much more easily and rapidly in rodents versus humans, duration of cooling, depth of cooling, differences in medical devices used to induce/maintain hypothermia across studies, rate of rewarming, managing adverse side effects, injury heterogeneity, differences in background care between rodents and critically ill patients, including risk of side effects such as bleeding, nosocomical infection, and concurrent drug use, existence of clinical confounders impacting data analysis, and, of course, species-specific differences.

**FIG. 1.**
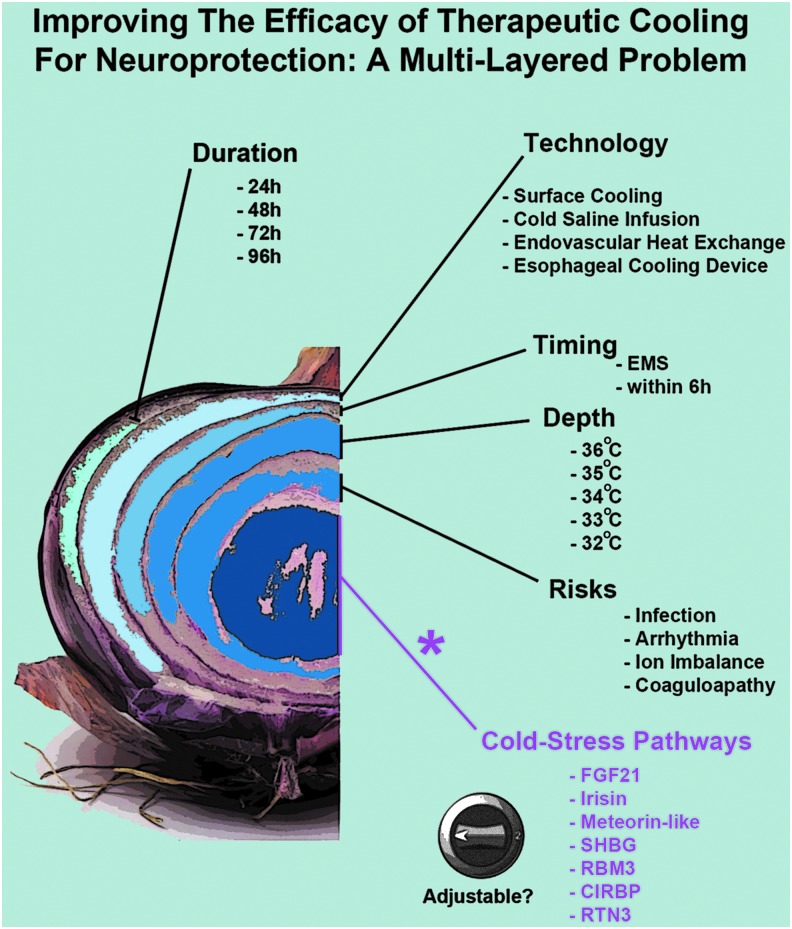
The many layers of cerebroprotective cooling: the “Responsivity of Cold Stress Pathways” is an additional (new) concept for optimizing TH. Multiple interdependent factors affect the efficacy of neuroprotective TH in patients. Major variables include the duration of cooling, the device/instrumentation used to induce hypothermia, the time to reach target temperature, the depth of cooling, and the prevention of detrimental side effects. *Purple text:* the optimal hypothermia protocol(s) that increase tissue/plasma levels of neuroprotective CSHs remain to be elucidated. Nor is it known if TH is able to increase CSPs in the brain in human adults. Age-dependent and other patient-specific differences may alter (increase or decrease) the induction of CSHs/CSPs by TH, which in turn may influence neurological recovery after a CNS injury. Furthermore, additional work is needed to determine if CSHs/CSPs can be optimized (i.e., adjusted) using noncooling interventions such as pharmacological approaches. CNS, central nervous system; CSHs, cold stress hormones; CSPs, cold shock proteins; TH, therapeutic hypothermia.

Here we propose adding, “Responsivity of Cold Stress Pathways,” to TH as another (new) factor that may influence the success of TH in clinical care (Fig. 1). It is the concept that an interaction of variables (e.g., age and/or other unidentified signaling processes) determines the magnitude by which hypothermia increases the levels/activation of cold stress molecules in biological systems. The goal of this review is to link emerging evidence in endocrinology, hibernation, neurocritical care, and brain injury research, often viewed in isolation, but together suggesting that cold stress pathways may alter brain physiology during TH and that they may need to be tailored to the individual to optimize the body's response to hypothermia. We begin with a brief overview of the classic neuroprotective mechanisms that are known to operate during TH, based on preclinical studies in models of brain injury and studies in patients, and also highlight the notable success of TH in humans for the treatment of HIE in infants. Next, to begin to link those traditional findings to the concept of “Responsivity of Cold Stress Pathways,” to TH, we review four cold stress hormones (CSHs) and three cold shock proteins (CSPs), discuss their prominent role in the developing brain, and speculate on the manner in which modulating their levels may be an important step in optimizing TH-mediated neuroprotection in adult humans to compensate for a limited Responsivity of Cold Stress Pathways. Specifically, we review data supporting the notion that baseline Responsivity of Cold Stress Pathways is markedly increased in infants versus adults (where it is nearly absent), and may contribute to the greater efficacy of neuroprotective TH in that population. Finally, we conclude with a discussion of “Hypothermia in a Syringe”—which is the concept that facets of neuroprotective cooling might be formulated into an intravenous (IV) infusion of molecules that induce molecular components of the cold stress response, increasing neuroprotective effectors despite maintaining the organism at 37°C. We envision a potential broad range of medical applications for Hypothermia in a Syringe, such as for the treatment of normothermic patients with chronic neurodegenerative diseases. However, to emphasize its potential applications in settings well beyond existent medical needs, and to envision the risks and benefits of this strategy under injury conditions that would affect the entire organism (brain and body), we close by discussing the futuristic possibilities of using Hypothermia in a Syringe to target cellular pathways that defend against the detrimental effects associated with long-duration spaceflight.

## Classic Mechanisms Mediating Neuroprotective Hypothermia

**FIG. 2.**
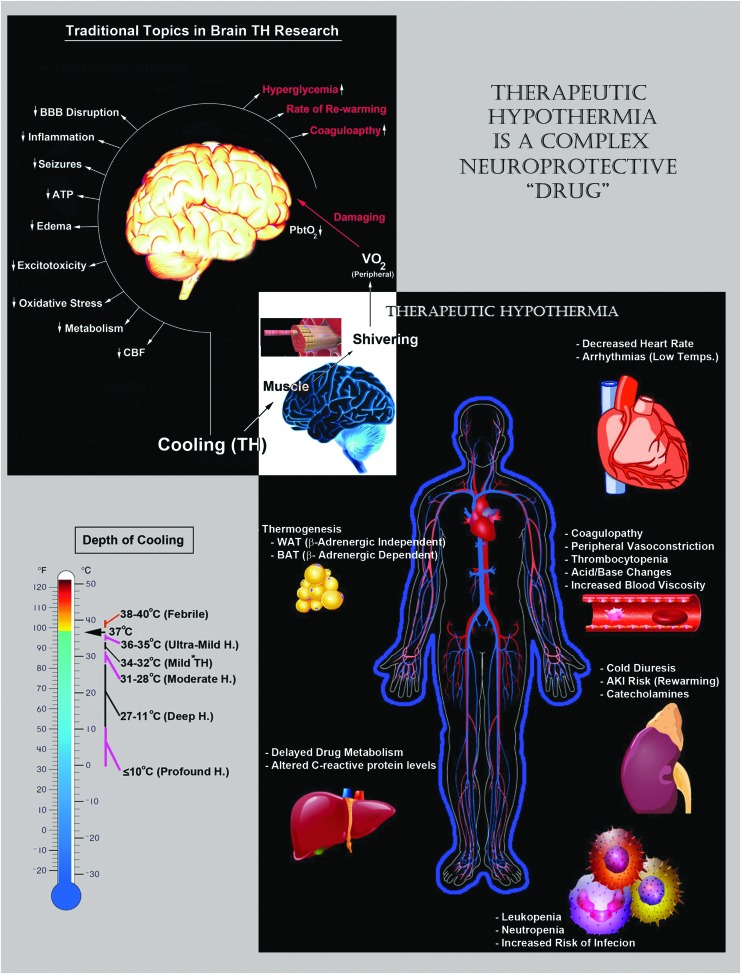
Important (classic) mechanisms of neuroprotective hypothermia and potential side effects of total body cooling. *Upper-left/white text*: a broad group of neuroprotective mechanisms mediate neuroprotection by cooling in the CNS. *Bottom*-*left*: the magnitude of induction of different neuroprotective mechanisms depends, in part, on the depth of cooling. Clinically, the temperature ranges are divided into mild, moderate, deep, and profound. Recently, the term UMH was introduced to include therapeutic temperatures ranging above >35°C and below <36°C. *Bottom*-*right*: total body cooling is a complex “drug” that affects almost every organ/tissue in the body. Maximizing the clinical benefits of cerebral cooling depends, in part, on monitoring/controlling adverse side effects of hypothermia, germane to functional changes in other organ systems, which may inadvertently pose a risk to patient survival and/or CNS recovery after an injury. UMH, Ultramild Hypothermia.

Clinically, the depth of hypothermia is stratified into mild (34–32°C), moderate (31–28°C), deep (27–11°C), and profound (<10°C) (Fig. 2, lower left). More recently (2015), we introduced the term Ultramild Hypothermia (UMH) for temperatures >35°C and ≤36°C based on evidence that (1) 36°C versus 37°C induced a bona fide intracellular cold shock response in cultured primary neurons *in vitro* (Jackson *et al.*, 2015), (2) Berntman *et al.* (1981) showed that 36°C versus 37°C improved biochemical markers of brain damage after hypoxic/ischemic injury in adult rats, and (3) as discussed earlier, rigorous clamping of patients at 36°C confers benefit after cardiac arrest, an effect that may not simply represent prevention of fever (Nielsen *et al.*, 2013).

Numerous mechanisms mediate the cerebroprotective effects of cooling. Although in this review we have focused on the potential role of CSPs/CSHs in acute brain injury and chronic neurodegeneration, it is important to briefly summarize the classic neuroprotective mechanisms induced by hypothermia, to provide a base of understanding, and to clarify the integration of novel concepts posed here into the full scheme of hypothermic neuroprotection (Fig. 2, upper left). Notably, some neuroprotective mechanisms provide increased benefit with deeper levels of cooling (e.g., decreased cerebral metabolic rate [CMR]), whereas others do not scale with temperature depth and may become inhibited at very low levels of hypothermia (e.g., CSPs—discussed later). Furthermore, modest differences in the target temperature can robustly alter if TH therapy is protective, or conversely, exacerbates damage after a brain injury (Alonso-Alconada *et al.*, 2015). Thus, the depth of hypothermia is an important factor that modulates the fractional contribution of each mechanism on the total cumulative benefits of cooling. These interactions and their link to specific levels of TH, along with different central nervous system (CNS) insults and insult severity among other factors, can be rather complex. However, for the purpose of this brief overview, we omit an expansive discussion on the optimal temperature(s) that may maximally target different (classic) neuroprotective mechanisms.

Brain ischemia mediates neuronal injury after a cardiac arrest or stroke, for example, and contributes to secondary injury after a TBI (particularly if comorbid with hypoxemia and/or hypotension) (Kaufmann *et al.*, 1999; Coles *et al.*, 2004; Hlatky *et al.*, 2004; Cunningham *et al.*, 2005; Iordanova *et al.*, 2017). The amount of ATP (cellular energy) that neurons require to survive is proportional to CMR. Ischemic cell death is caused by an imbalance in ATP supply (i.e., hypoxia-mediated loss of oxidative phosphorylation) versus demand (high oxygen consumption [CMRO_2_] of brain tissue). Germane to TH, each 1°C drop in brain temperature (37–27°C) decreases CMRO_2_ ∼6–7% (Edgar and Bering, 1961; Steen *et al.*, 1983). Therefore, hypothermia can limit or prevent the development of ischemia during episodes of disturbed or severely compromised CBF by decreasing the ATP utilization and oxygen tension thresholds required to sustain tissue viability (Metz *et al.*, 1996).

Other derangements in CBF physiology are also improved and/or impacted by hypothermia, but their role in mediating hypothermic protection is much more complex, less compelling, and less well understood. For instance, after a TBI, delayed hyperemia (luxury perfusion) exacerbates vasogenic tissue edema and promotes intracranial hypertension (intracranial pressure [ICP] > 20 mmHg) in brain regions that have lost cerebral autoregulation (Obrist *et al.*, 1984). Treatment with hypothermia decreased reactive hyperemia after reperfusion in a feline balloon inflation model of severe intracranial hypertension (i.e., ICP was raised to a level that produced brain ischemia) (Mori *et al.*, 1998). The mechanism of protection may have involved decreased CMR (with cooling) and a resultant decrease in CBF due to metabolic/flow coupling. In addition, hypothermia decreased cytotoxic edema formation by downregulating brain aquaporin 4 (AQP4) water channels in a model of cerebral ischemia/reperfusion injury, which would also help lower ICP and improve CBF (Kurisu *et al.*, 2016). Conversely, hypothermia increased membrane levels of AQP4 in astrocytes *in vitro* (Salman *et al.*, 2017). This type of complexity in response to TH mandates the need for additional studies to clarify if cooling the brain promotes CNS recovery by targeting edema and/or high ICP in certain patients. However, a recent RCT reported that TH failed to improve long-term neurologic outcomes in a severe TBI cohort of adults with intracranial hypertension (Andrews *et al.*, 2015; Flynn *et al.*, 2015).

Oxidative stress also mediates tissue damage after a brain injury and is a mechanism where there is substantial support for a role of TH in limiting its deleterious consequences. It results from increased production of toxic oxidizing agents (reactive oxygen species [ROS] or reactive nitrogen species [RNS]) versus decreased capacity of intracellular scavenging mechanisms to neutralize oxygen (O_2_^•−^, OH^•^, and H_2_O_2_), nitrogen (ONOO^−^), and additional radical intermediates via antioxidant defenses (Kohen and Nyska, 2002). Oxidative damage in the CNS after an acute injury, or in chronic neurodegenerative disease, has been extensively reviewed elsewhere (Chen *et al.*, 2011a; Cornelius *et al.*, 2013; Tonnies and Trushina, 2017). There are numerous ROS and/or RNS generating mechanisms, including disruption in the mitochondrial respiratory chain, activation of enzymes that promote ROS (e.g., xanthine oxidase, NADPH oxidase), and redox cycling agents (e.g., free iron), among others (Kohen and Nyska, 2002). The cumulative effect is direct damage (oxidation) to proteins, lipids, and RNA/DNA (Kohen and Nyska, 2002). Moreover, depletion of endogenous antioxidants after an injury (e.g., consumption of reduced glutathione) further exacerbates oxidative stress (Kohen and Nyska, 2002). TH inhibits oxidative brain damage by decreasing these generating processes (Lei *et al.*, 1994, 1997; Globus *et al.*, 1995; Kil *et al.*, 1996; Chatzipanteli *et al.*, 1999) and limiting consumption of antioxidant defenses, shown in both preclinical models and in patients across a number of CNS insults (Bayir *et al.*, 2009; Hackenhaar *et al.*, 2017).

Excessive neuronal depolarization after a brain injury results in intracellular Ca^2+^ overload and sustained glutamate release (excitotoxicity) (Chamoun *et al.*, 2010; Schober *et al.*, 2016). Moreover, extracellular glutamate levels are further increased by pathological reversal of astrocytic glutamate transporters (Gouix *et al.*, 2009). The cumulative effect is rapid activation of extrasynaptic N-methyl-D-aspartate (NMDA) receptors (NMDARs), triggering intracellular apoptotic/necrotic signaling cascades leading to neuronal death (Hardingham *et al.*, 2002). In addition, synaptic NMDARs promote hypoxic cell death or (conversely) stimulate neuroprotective pathways (Hardingham and Bading, 2010; Wroge *et al.*, 2012). TH potently inhibits neuronal death induced by direct intraparenchymal injection of glutamate into the brain (Suehiro *et al.*, 1999). Furthermore, cerebral cooling prevents postinjury spikes in extracellular glutamate in models of ischemia (Mitani and Kataoka, 1991; Ooboshi *et al.*, 2000; Campos *et al.*, 2012), fluid percussion TBI (Globus *et al.*, 1995), subarachnoid hemorrhage (Shuaib *et al.*, 1996; Schubert *et al.*, 2008), and bacterial meningitis (Irazuzta *et al.*, 1999a). In contrast, cooling had no effect on postinjury glutamate levels in a model of controlled cortical impact (CCI), TBI but was cerebroprotective (Palmer *et al.*, 1993). Finally, hypothermia (32°C) paradoxically increased postinjury glutamate levels above normothermic controls in a rat weight-drop contusion model of TBI (Koizumi *et al.*, 1997). Thus, cooling potently blocks glutamate-mediated neuronal death in many (but not all) brain insult types. Also, concerns have been raised that targeting excitotoxicity may have limited efficacy in brain-injured patients due to the transient therapeutic time window available to prevent the intracellular catastrophe induced by Ca^2+^/glutamate overload. This concern may be particularly important for TH due to the technical challenges involved in initiating cooling in critically ill patients, and the resulting time delay to reach target temperature. Indeed, in a rat cardiac arrest model, brief TH (31°C) blocked glutamate release if applied before ischemia or at the time of return of spontaneous circulation (ROSC), but was ineffective if initiated ∼5 minutes postresuscitation (Takata *et al.*, 2005). Similarly, in a dog ventricular fibrillation (VF) cardiac arrest model, the beneficial effects of mild TH (34°C) on neurological outcome and survival were negated if cooling was delayed 20 minutes postresuscitation (Nozari *et al.*, 2006). Finally, cerebral cooling also shows great promise to prevent early/chronic seizures (e.g., refractory status epilepticus or cortical spreading depolarization), which similarly arise, in part, from dysregulation of glutamatergic neurotransmission, but have a much broader time window for therapeutic intervention after a brain injury (Takaoka *et al.*, 1996; Corry *et al.*, 2008; Hartings *et al.*, 2009; Barker-Haliski and White, 2015; Niquet *et al.*, 2015; Schiefecker *et al.*, 2018).

Excitotoxic and oxidative injury mechanisms initiate a cascade of events, which lead to a potent neuroinflammatory response—the molecular underpinnings of inflammation in the brain were recently reviewed by our group (Simon *et al.*, 2017). Liberation of intracellular DNA and debris from dying cells into the extracellular space, and release of additional damage-associated molecular patterns (DAMPs), triggers an immediate (within minutes/h) proinflammatory cytokine response in the brain (e.g., increased TNF, IFNγ, and IL-6) (Frugier *et al.*, 2010; Ansari, 2015). Moreover, neutrophils rapidly accumulate in the early acute phase following a TBI, and after the reperfusion phase in cerebral ischemia (Garcia *et al.*, 1994; Carlos *et al.*, 1997; Price *et al.*, 2004).

Later, evolving time-dependent changes in the release of additional downstream cytokines/chemokines, and in response to neuronal death, alters the extracellular milieu days/weeks postinjury and initially promotes a proinflammatory (M1/M1-like) phenotype in infiltrating macrophages and resident microglia (Harting *et al.*, 2008; Hu *et al.*, 2012; Boddaert *et al.*, 2018). In the chronic phase, ideally, macrophages and microglia switch to an anti-inflammatory (M2/M2-like) phenotype, promoting wound healing and injury resolution. However, recent findings suggest that M2/M2-like responses peak in the subacute/early chronic phase after a TBI, followed by a prolonged and detrimental shift toward an M1/M1-like phenotype (Loane and Kumar, 2016). Furthermore, the evolving trajectory of pro- versus anti-inflammatory functions of immune cells differs among individuals (e.g., by insult mechanism, tissue type [white vs. gray matter], and additional factors effecting patient heterogeneity), and a significant fraction of severe TBI victims develop chronic/persistent neuroinflammation that temporally and spatially matches progressive axonal injury (Ramlackhansingh *et al.*, 2011). TH decreases neuroinflammation by blocking its triggers (e.g., preventing cell death mechanisms). In addition, cooling the brain shifts monocytes toward an anti-inflammatory M2 phenotype (Truettner *et al.*, 2017; Liu *et al.*, 2018). However, in some instances, cooling might aggravate inflammation. Indeed, TH in piglets precipitated a proinflammatory cytokine surge after rewarming versus normothermic controls, and underscores the need for additional research to define the optimal rewarming protocol(s) in different patient cohorts (Rocha-Ferreira *et al.*, 2017).

The blood/brain barrier (BBB) maintains the chemical composition of the brain interstitial fluid and is critical for normal CNS function (Sweeney *et al.*, 2019). Increased BBB permeability due to mechanical disruption after a TBI, or by other disease processes, increases extravasation of harmful pathogens and toxic micro/macro molecules (e.g., bilirubin) into the underlying brain parenchyma (Wennberg and Hance, 1986; Kristensson, 2011). Also, leakage of erythrocytes into the perivascular space, followed by subsequent hemolysis, results in increased extracellular hemoglobin and free iron, exacerbating ROS-mediated injury (Rifkind *et al.*, 2014). TH decreased BBB damage in models of head trauma (Smith and Hall, 1996; Lotocki *et al.*, 2009), stroke (Tang *et al.*, 2013; Liu *et al.*, 2017), bacterial meningitis (Irazuzta *et al.*, 1999b, 2000), and intracerebral hemorrhage (Song *et al.*, 2018), among others. The mechanisms of hypothermia-mediated protection of the BBB involve inhibition of matrix metalloproteinases (Lee *et al.*, 2005), preservation of tight-junction proteins (Li *et al.*, 2017b), and downregulation of intracellular adhesion molecule 1 on the surface of vascular endothelium, preventing leukocyte diapedesis (Lotocki *et al.*, 2009; Choi *et al.*, 2011b). Maintaining BBB integrity is also important to maximize the benefits of hyperosmolar therapies used to decrease brain water content/edema and prevent possible “rebound ICP” in the treatment of intracranial hypertension (Torre-Healy *et al.*, 2012).

Activation of upstream insults (reviewed above) stimulates a diverse set of downstream cell signaling pathways, which engage different types of cell death mechanisms and collectively result in secondary brain injury. Each cell death mechanism has its own unique “molecular signature,” which involves specific effector molecules and signaling cascades. A comprehensive review of the manner in which hypothermia alters individual components of each cell death pathway is outside the scope of this focused article. In general, there is abundant evidence across a spectrum of brain injury models demonstrating that mild/moderate hypothermia inhibits either the protein levels and/or activation of molecular targets that mediate apoptosis (Edwards *et al.*, 1995; Gong *et al.*, 2013; Eroglu *et al.*, 2017), necrosis (Buki *et al.*, 1999; Liebetrau *et al.*, 2004), autophagy (Lu *et al.*, 2014; Song *et al.*, 2018), necroptosis (Liu *et al.*, 2016), or pyroptosis (Tomura *et al.*, 2012; Zhou *et al.*, 2018). Conversely, profound hypothermia can decrease tissue viability, and the mechanisms mediating this phenomenon are under investigation; Hattori *et al.* (2017) reported that temperatures less than ∼10–8°C robustly activate ferroptosis in a broad range of cell types *in vitro* (i.e., Fe^+^/MAPK-dependent and lipid peroxidation-mediated cell death). A greater understanding of cell death mechanisms activated by deep or profound hypothermia may improve the efficacy of treatments such as deep hypothermic circulatory arrest (DHCA), in which longer surgery time is well-known to increase the risk of cognitive impairment post-resuscitation (Kumral *et al.*, 2001).

Finally, mitigating adverse side effects of TH is also critical for improving the clinical efficacy of cooling in the treatment of brain injury (Fig. 2, bottom right). Key health risks include coagulopathy, increased rates of infection, intense shivering (which may activate a stress response and increase metabolic demands), arrhythmias, and hyperglycemia, among others (Noyes and Lundbye, 2015). In general, these adverse events are exacerbated by greater depths of cooling but can typically be well managed at temperatures spanning the mild TH range (Polderman, 2009).

## The Influence of Developmental Age on the Efficacy of TH in Humans

Developmental age robustly alters gene expression, protein levels, epigenetic/post-transcriptional modifications, and connectivity in the brain (Cheung *et al.*, 2010; Colantuoni *et al.*, 2011; Lipovich *et al.*, 2014; Walhovd *et al.*, 2016). A similar CNS insult (e.g., mechanism and magnitude) may elicit different cell signaling and biochemical responses in damaged tissue from patients at different ages (e.g., toddlers vs. adults). In addition, patient age is a major factor that affects the efficacy of neuroprotective therapies in clinical trials (e.g., NIH's “Inclusion Across the Lifespan Policy”), and suggests that the benefits of an intervention depend, in part, on the unique molecular framework at a given age versus the extent that a treatment is optimal for that particular time point. For instance, caspases and other proapoptotic proteins are abundant in the newborn brain and facilitate homeostatic pruning of surplus synapses/dendrites (Shimohama *et al.*, 2001; Soane *et al.*, 2008; Yamaguchi and Miura, 2015). Following CNS injury, increased baseline expression of caspases in the infant brain can be maladaptive and indiscriminately enhance apoptotic cell death in otherwise salvageable tissue. Therapies that block caspase-mediated cell death are particularly robust in models of developmental brain injury (Li *et al.*, 2000; Han *et al.*, 2002; Chauvier *et al.*, 2011). Numerous additional examples include age-dependent vulnerabilities and/or differences in oligodendrocytes (Salter and Fern, 2005), antioxidant defenses (Ditelberg *et al.*, 1995; Nanda *et al.*, 1996; Fan *et al.*, 2003), and changes in excitatory neurotransmission that affect the magnitude of Ca^2+^ influx through voltage/ligand-gated ion channels (Ramoa and Mccormick, 1994; Zhou and Baudry, 2006; Henson *et al.*, 2008), among others.

The neuroprotective efficacy of TH is also robustly influenced by age. The cerebroprotective effects of TH in humans are greatest in newborns, based on meta-analysis of clinical trials in moderate/severe HIE, and seven is the number needed to treat to observe one beneficial outcome (NNTB) on combined mortality or long-term neurodevelopmental disability (Jacobs *et al.*, 2013). However, the efficacy of TH in the setting of mild HIE is unclear, and currently is not recommended as standard of care in advance of additional research (Kariholu *et al.*, 2018). In contrast, recent trials in adults and children with cardiac arrest or TBI reported that patients managed with TH (33°C for ∼24–48 hours) had similar long-term neurologic outcomes versus those given standard of care (TTM/fever prevention) (Adelson *et al.*, 2013; Nielsen *et al.*, 2013; Maekawa *et al.*, 2015; Moler *et al.*, 2015, 2017; Cooper *et al.*, 2018). The biological underpinnings mediating age-dependent discrepancies in the efficacy of neuroprotective TH have not been fully elucidated. Identifying the relevant cell signaling pathways (1) may lead to novel approaches to augment the efficacy of TH in newborns (i.e., an NNTB of 7 indicates that many newborns with moderate/severe HIE are not protected by cooling), (2) aids development of biomarker-based tests to predict which patients will benefit most from cooling, and (3) determines if adults lack key molecular substrates expressed in the young that (in part) mediate neuroprotective effects of TH and thus represent a viable target to enhance the efficacy of TH across the spectrum of CNS injuries.

In addition, germane to both age and species, results of rodent brain injury models have tended to overestimate the anticipated success of TH in human adults. Indeed, brain injury studies in rats and mice indicate that newborn and adults are similarly (robustly) protected by cerebral cooling (contrary to findings in human trials) (Dietrich, 2000; van der Worp *et al.*, 2007). Animal homogeneity (e.g., inbred rodent strains) and the time it takes to initiate cooling, which can be achieved almost immediately in the laboratory, are a few of the potential factors contributing to discrepancies in preclinical versus clinical findings (Nozari *et al.*, 2006; Rocha-Ferreira *et al.*, 2018). However, species-specific differences likely play a role as well (Mestas and Hughes, 2004; Ellenbroek and Youn, 2016; Montenegro *et al.*, 2016; Reitman, 2018). One strategy for improving the efficacy of TH in adult humans is to elucidate which classic neuroprotective mechanisms might be better targeted by cooling in rodents versus humans, and then determine if it is feasible to transiently “rewire” human physiology to better match rodents to maximize the benefits of cooling, or augment TH with combination therapy with a drug or drugs targeting those mechanisms. An alternative and more novel strategy is to find neuroprotective cold-induced mechanisms that are poorly activated by TH in both adult rodents and in humans (i.e., highly conserved maladaptive physiology). Novel drug/treatments able to reanimate a latent/“resistant” cold-induced neuroprotective mechanism in rodents may then have an opportunity to translate into patients due to conserved biology. This implies that the full potential of neuroprotective TH has not yet been realized even in adult rodents (despite its well-known efficacy), given that the full spectrum of beneficial pathways has not been engaged. We think that CSPs, and in particular RNA binding motif 3 (RBM3; discussed below), are good examples of conserved TH-regulated targets that have not been optimized in either adult rodents or in humans. The sections that follow explore the utility of CSHs and CSPs to improve the efficacy of TH in humans, and this concept is reviewed to a large extent independent of the age(s) at which targeting these pathways may work best. Nevertheless, accumulating evidence suggests that baseline levels/activity of CSHs/CSPs are increased in newborns (rodents and humans) and thus represent a key age group for initial exploration germane to their therapeutic manipulation.

## The CSH Response During Cold Stress: Potential Applications for Neuroprotection

**FIG. 3.**
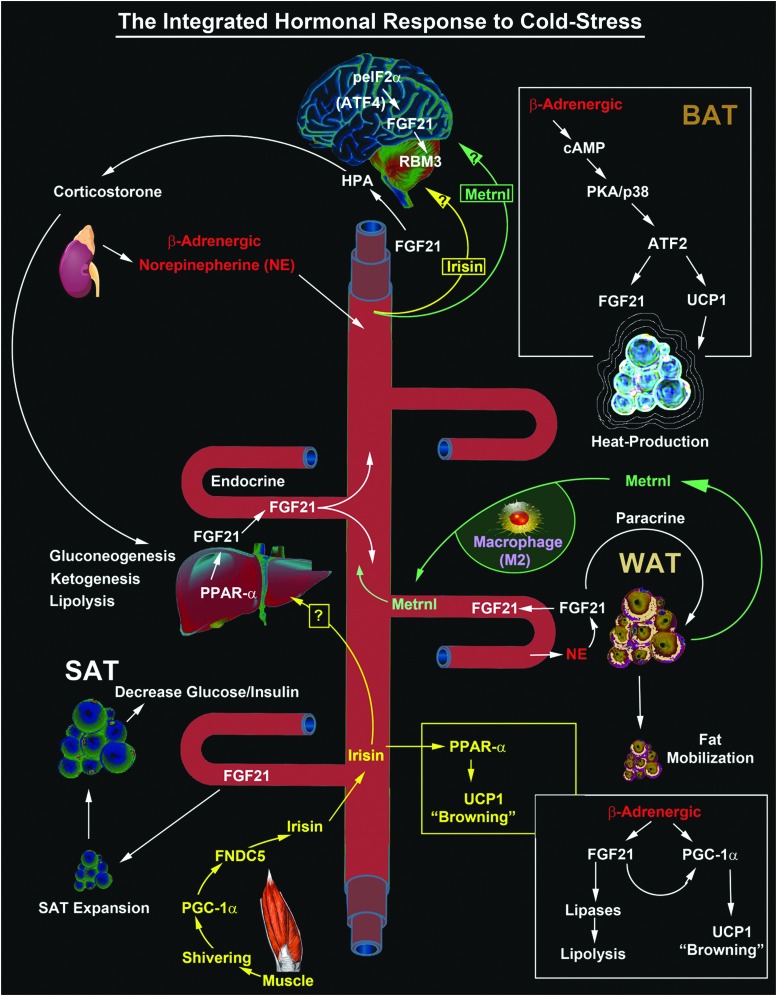
The complex interplay/release of cold stress hormones (FGF21, Irisin, and Metrnl) by thermogenesis-regulating organs after cold exposure and possible targeting to the brain. The diagram shows the major sources of key circulating CSHs. FGF21-regulated mechanisms are illustrated in *white text*. Irisin-regulated mechanisms are illustrated in *yellow text*. Metrnl-regulated mechanisms are illustrated in *green text*. Potential unknown intersections (?) of paracrine effects on target tissues are indicated. All known signaling links and molecular targets are supported by research articles cited in the primary text. ATF, activating transcription factor; BAT, brown adipose tissue; eIF2α, eukaryotic initiation factor 2-alpha; FGF21, fibroblast growth factor 21; Metrnl, Meteorin-like; PKA, protein kinase A; PPARα, peroxisome proliferator-activated receptor-alpha; RBM3, RNA binding motif 3; SAT, subcutaneous adipose tissue; UCP, uncoupling protein; WAT, white adipose tissue.

**FIG. 4.**
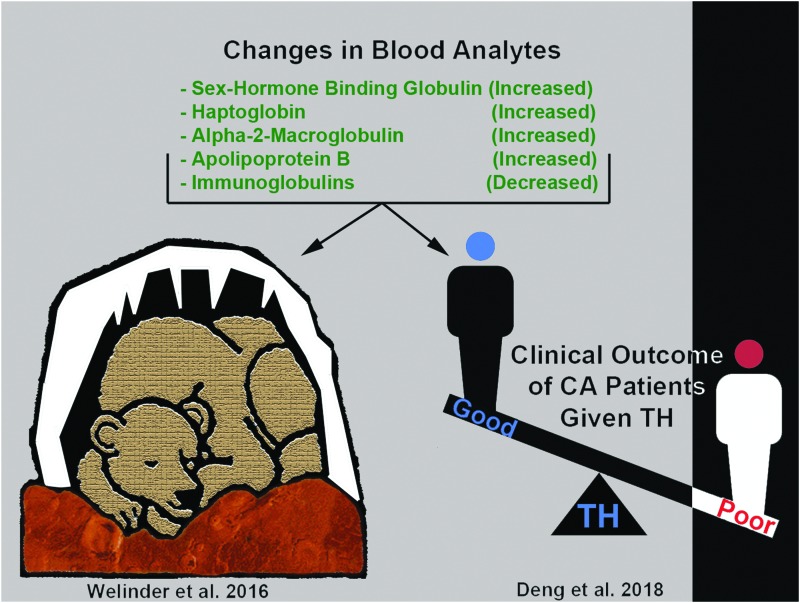
SHBG is a novel target of hypothermia with unknown function(s) postcooling in humans and in bears. Illustration shows protein targets that are similarly altered (increased or decreased) by cooling in juvenile hibernating bears (Welinder *et al.*, 2016) versus adult human CA patients treated with TH and who had a good neurological outcome (Deng *et al.*, 2018). In both studies, proteomic changes were detected by mass spectrometry of blood plasma, and SHBG levels were among the highest fold change (compared with respective controls) among the identified proteins. CA, cardiac arrest; SHBG, sex hormone binding globulin.

Beyond the aforementioned classic acute neuroprotective mechanisms of TH/TTM, a cascade of mechanisms and events is also linked to the use of TH that is mediated by CSHs and CSPs. CSHs activate thermogenic pathways and help to maintain core body temperature (T_b_). Emerging evidence suggests that CSHs evoke a broad set of molecular and biochemical changes, which may boost neuroprotective cooling. Brain researchers have only begun to study the involvement of CSHs on neurological outcomes in the setting of TH/TTM. The increased interest may relate to underwhelming results of clinical trials on TH in adult human studies, compared with the highly compelling data obtained by preclinical animal experiments, and a shift in thinking leading to a renaissance or return to focus on understudied cell signaling pathways affected by systemic cooling, which might directly or indirectly impact the brain. Here we review four CSHs that may represent low-hanging fruit for additional investigation as to their potential as novel agents to augment hypothermic neuroprotection in neurocritical care, or to decrease neuropathology in normothermic patients with chronic neurodegenerative diseases (Figs. 3 and 4).

### Fibroblast growth factor 21

Most fibroblast growth factors (FGFs) are paracrine hormones (reviewed elsewhere; Itoh and Ornitz, 2008; Ornitz and Itoh, 2015). A conserved heparin-sulfate proteoglycan binding domain (HSPBD) restricts their activity to near the site of release (Thompson *et al.*, 1994). In contrast, FGF21 is a member of the endocrine subfamily of FGFs. Endocrine hormone FGFs (FGF19, FGF21, and FGF23) lost their heparin binding function during evolution causing them to circulate freely on release (Itoh and Ornitz, 2008). Consequently, they utilize transmembrane klotho proteins as coreceptors (α-klotho and/or β-klotho), which function as molecular scaffolds to promote/stabilize the interaction between extracellular ligand and tissue receptor (Chen *et al.*, 2018a; Lee *et al.*, 2018b). β-klotho is the obligatory coreceptor for FGF21 and is required for ligand binding and activation of FGF1Rc *in vivo* (Kurosu *et al.*, 2007; Adams *et al.*, 2012). Also, *in vitro* studies show that β-klotho increases the affinity of FGF21 to bind multiple FGF receptor isoforms, but the magnitude of activation of downstream targets varies by receptor type (FGFR1c >FGFR2c >FGFR3c) (Kurosu *et al.*, 2007). Furthermore, β-klotho expression in adults is limited to the liver, pancreas, adipose tissue, and a few neuronal populations within the hypothalamus/hindbrain (Tacer *et al.*, 2010; Bookout *et al.*, 2013). The near absence of β-klotho messenger RNA (mRNA) in most regions of the adult brain has been confirmed in mice and in 13-lined ground squirrels (Tacer *et al.*, 2010; Bookout *et al.*, 2013; Nelson *et al.*, 2013). Also, our group confirmed that β-klotho protein levels are negligible in the frontal cortex and in the hippocampus of human adolescent or adult subjects (Jackson *et al.*, 2018). In contrast, β-klotho is unexpectedly abundant in the cortex and in the hippocampus in infants, and is also expressed in toddlers (Jackson *et al.*, 2018). The potential importance of this discovery is addressed later.

FGF21 is an integral hormone in the mammalian metabolic response to cold stress. T_b_ affects FGF21 levels and vice versa. Inagaki *et al.* (2007) were the first to report that FGF21 has a direct effect on T_b_. FGF21 mRNA overexpression in the liver induced torpor on 24 hours of fasting in transgenic mice (Inagaki *et al.*, 2007). During the 12-hour-light cycle phase of the experiment, T_b_ was 1–2°C lower in fasted transgenic mice versus wild types. During the 12-hour-dark cycle phase, T_b_ plummeted to <28°C in fasted transgenic mice, which became physically inactive, whereas T_b_ in wild-type mice remained ≥34°C and torpor was not induced. Also, the absence of torpor in fed transgenic mice suggests that FGF21 pathways interact with starvation-stimulated signaling mechanisms, which together coordinate decreased T_b_. The FGF21 transgene was under control of an APOE promoter, which caused an ∼50-fold increase in FGF21 mRNA levels in the liver of transgenic versus wild-type mice (Inagaki *et al.*, 2007). The brain is the second highest expresser of APOE, and it is abundant in astrocytes, cells of the choroid plexus, and in smooth muscle surrounding CNS blood vessels (Srivastava *et al.*, 1996; Xu *et al.*, 2006). Thus, presumably, brain FGF21 levels also greatly increased in transgenic mice during the fasting period. The contribution of brain-derived FGF21 to affect torpor/T_b_ in this study, potentially by acting on β-klotho expressing neurons in the hypothalamus, was not explored.

Nelson *et al.* (2013) directly tested the hypothesis that peripherally derived FGF21 is a key inducer of hibernation in 13-lined ground squirrels. Adenovirus-mediated FGF21 overexpression, delivered via the femoral artery, failed to increase the number of squirrels entering torpor during simulated hibernation (fasting/24-hour darkness/5°C for 7 days). Interestingly, FGF21 overexpression decreased the mean-minimum T_b_ in active squirrels maintained at normal ambient temperature (23°C) but conversely increased total mean T_b_ in squirrels subjected to simulated hibernation; potentially due to increased thermogenesis in the latter (Nelson *et al.*, 2013). Thus, FGF21 modifies facets of T_b_ regulation (up or down) depending on the season, environmental conditions, and thermoregulatory need. Also, naive squirrels had very low endogenous blood FGF21 levels during active months, but the levels increased during winter torpor and were maximal (approximately eight- to ninefold higher vs. active months) during interbout arousals (IBAs), which are brief 24-hour periods of rapid rewarming followed by re-entering hypothermic torpor for 10 days (Nelson *et al.*, 2013). Whether the robust increase of FGF21 during IBA (1) is strictly related to the activation of heat producing thermogenesis, (2) protects animals against the negative aspects of rapid rewarming, or (3) activates cold adaptive signaling programs that improve organ health during deep hypothermia is unknown. Thus, the work of Nelson *et al.* (2013) did not establish that FGF21 is a key inducer of hibernation, but supports the notion that FGF21 is involved in the mammalian defense and/or tolerance to hypothermia.

Circulating FGF21 levels increase in rodents and in humans during cold stress. Lee *et al.* (2013) were the first to show this in humans. Patients subjected to a mild environmental cold challenge of 19°C for 12 hours, wearing only hospital scrubs, had increased plasma FGF21 levels versus subjects maintained under thermoneutral conditions (24°C). The same research team later confirmed these findings in a separate cohort of human subjects (Lee *et al.*, 2014). Of note, blood FGF21 levels are greatly affected by diurnal rhythms in humans; plasma levels are highest in the early morning and then decrease to a nadir in late afternoon (Yu *et al.*, 2011; Lee *et al.*, 2013, 2014). Cold stress blunts the diurnal decrease but does not prevent it (Lee *et al.*, 2013, 2014). Thus, future studies should take into account the time of day samples are collected, and consider the impact of diurnal changes across comparison groups if serum FGF21 levels are measured in brain-injured patients.

Cold stress also increases blood FGF21 levels in rodents. Chartoumpekis *et al.* (2011) showed in mice that a decreased environmental temperature to 4°C for 4 hours robustly increased FGF21 mRNA levels in brown adipose tissue (BAT), however, plasma FGF21 protein levels were not elevated at that time point. Hondares *et al.* (2011) showed that mice housed at 4°C (environmental temperature) for 6 hours, 24 hours, or 30 days had increased FGF21 mRNA levels in BAT at all three time points. Plasma FGF21 levels were not elevated by 6 hours of cooling but increased by 24 hours, and were markedly increased by chronic cooling for 30 days. Thus, cold stress increases FGF21 expression in BAT in mice, and, after a subacute delay (<1 day), leads to increased circulating FGF21 protein levels in the blood.

What are the intracellular signaling mechanisms mediating increased FGF21 levels during cold stress and what are the downstream physiological consequences of FGF21 receptor activation? The majority of experiments addressing these questions used tissues/cells, which constitutively express β-klotho in adults (i.e., liver, pancreas, adipose). Thus, most information on FGF21 signaling mechanisms may be biased to understanding the function in those organs; therefore, we refer to this as the “canonical pathway” for the sake of this review. We speculate that additional FGF21 signaling mechanisms are yet to be brought to light, such as in tissues like the infant hippocampus or cerebral cortex, also expressing β-klotho, but in which we would not expect the role of FGF21 to be increased stimulation of intracellular lipolysis or enhanced heat production, as is the case for organs involved in thermal homeostasis.

In the liver, FGF21 gene expression is regulated by the activation of nuclear peroxisome proliferator-activated receptor alpha (PPARα), the latter is powerfully induced by starvation (Inagaki *et al.*, 2007). Cold shivering also increases the circulating hormone irisin, which stimulates nuclear PPARα in white adipose tissue (WAT; discussed in greater detail in the next section) (Bostrom *et al.*, 2012). To our knowledge it has not been tested if increased blood irisin levels activate PPARα in the liver; however, it could theoretically further stimulate FGF21-mediated cold stress mechanisms (i.e., muscle shivering induced release of hepatic FGF21). In BAT, FGF21 gene expression is regulated by cold-induced activation of the sympathetic nervous system, resulting in increased catecholamine release (e.g., norepinephrine), in turn activating adrenergic receptors, which stimulates activating transcription factor 2 (ATF2) (Chartoumpekis *et al.*, 2011; Hondares *et al.*, 2011). Inhibition of PPARα in BAT does not block cold-induced or β-adrenergic agonist-induced FGF21 expression (Chartoumpekis *et al.*, 2011; Hondares *et al.*, 2011).

Starvation and/or cold stress increase the activation of FGFR1c/β-klotho signaling complexes in the liver, pancreas, adipose (BAT, WAT, and subcutaneous adipose tissue [SAT]), and hypothalamus. The broad physiological effects of FGF21 on metabolism have been comprehensively reviewed elsewhere (Fisher and Maratos-Flier, 2016). In the liver, FGF21 promotes ketogenesis (synthesis of ketone bodies), lipolysis, and stimulates gluconeogenesis (Inagaki *et al.*, 2007; Liang *et al.*, 2014). In WAT, FGF21 increases the expression of lipases that mobilize fat stores, and also increases enzymes that catabolize fatty acids to make acetyl-CoA (Inagaki *et al.*, 2007; De Sousa-Coelho *et al.*, 2013). Furthermore, in WAT, FGF21 increases uncoupling protein 1 (UCP1) levels via a post-transcriptional mechanism, which promotes browning and heat generation during cold adaptation (Fisher *et al.*, 2012). In BAT, FGF21 acts to increase glucose clearance and to sensitize insulin signaling (Kwon *et al.*, 2015; BonDurant *et al.*, 2017). More recently, it was discovered that FGF21 increased the expansion of SAT, which appeared to partially mediate its beneficial insulin/glucose-lowering effects (Li *et al.*, 2018); intriguingly, germane to cold adaptation, SAT thickness determines the extent to which adult humans can withstand extremely cold water and maintain normal T_b_ (Hayward and Keatinge, 1981). Thus, FGF21-mediated stimulation of SAT expansion may serve a dual purpose by promoting cold tolerance. Furthermore, the relationship between FGF21 levels and SAT in newborns merits additional investigation. Healthy human infants have high levels of circulating FGF21 in the first year of life, presumably due to increased PPARα-mediated expression in the liver based on animal studies in rodent neonates, and also, SAT thickness during the same growth period in human babies positively correlates with rates of motor development (Hondares *et al.*, 2010; Kanazawa *et al.*, 2014; Sanchez-Infantes *et al.*, 2015). In the pancreas, FGF21 represses growth hormone (GH)-mediated synthesis and the release of insulin from islet cells (So *et al.*, 2015). Finally, in the brain, FGF21 stimulates the hypothalamic/pituitary/adrenal (HPA) axis, which in turn stimulates gluconeogenesis in the liver via corticosterone (Liang *et al.*, 2014).

Evidence suggests that increased FGF21 levels may improve brain health after an acute CNS injury or in chronic neurodegenerative conditions. We consider the evidence to support both direct and indirect mechanisms of CNS benefit. Also, germane to the clinical practicalities of drug administration for brain-targeted therapies, it is highly desirable that FGF21 has been shown to cross the BBB. Radiolabeled ^125^I-FGF21 is detected in cortical brain tissue 10 minutes after IV injection and reaches the brain parenchyma by simple diffusion (Hsuchou *et al.*, 2007). Furthermore, in order for FGF21 to activate direct mechanisms of neuroprotection, β-klotho must be present in brain regions targeted by the therapy, such as in the hippocampus, a brain region that is known to be highly vulnerable to ischemic, traumatic, and other insults. In adults, this appears to be a major limitation to the potential utility of FGF21 as a neuroprotectant, because β-klotho is restricted to the hypothalamus/hindbrain. In contrast, we have reported that in infants and in toddlers, β-klotho expression is more widespread, including in the cortex and in the hippocampus (Jackson *et al.*, 2018). Thus, very-young brain-injured patients could be a key group that might benefit most from the direct neuroprotective effects of FGF21. Surprisingly, the distribution of β-klotho among different cell types in the infant brain is unknown (e.g., neurons, astrocytes, microglia, oligodendrocytes, pericytes, or endothelial cells). We are currently addressing this knowledge gap via ongoing experiments on human tissues.

FGF21 is directly neuroprotective. Leng *et al.* (2015) showed that 6 days pretreatment with 5 nM FGF21 decreased subsequent cell death induced by glutamate toxicity in immature day *in vitro* (DIV) 6 primary rat cortical neurons maintained at 37°C. FGF21 treatment also increased phosphorylation of neuronal AKT, ERK, and GSK-3β (Leng *et al.*, 2015). We reported that FGF21 augments the induction of the neuroprotective CSP RBM3 after 24 hours UMH to 36°C in DIV6–7 primary rat cortical neurons (Jackson *et al.*, 2015); interestingly, this synergistic effect was not observed in mature DIV26 neurons treated with FGF21 at 36°C. Kuroda *et al.* (2017) reported that peripherally derived FGF21 in adult mice promoted CNS remyelination after lysophosphatidylcholine (LPC)-induced injury/demyelination in the brain and in the spinal cord white matter; the mechanism of protection involved increased β-klotho expression in oligodendrocyte precursor cells, which was induced by LPC injury. Amiri *et al.* (2018) showed *in vitro* that FGF21 pretreatment decreased neuronal death of human neuronal SHSY5Y cells injured by Aβ_1–42_—directly linking to the possible benefits of FGF21 therapy in the setting of Alzheimer's disease. Chen *et al.* (2018b) showed that administration of recombinant FGF21 in adult normothermic mice increased BBB integrity, decreased brain edema and histological damage, and ameliorated neurological deficits after a CCI-TBI. Given that β-klotho is absent in brain regions damaged by a CCI-TBI *in vivo*, it is unclear if the benefits were due to the direct activation of unidentified FGF21-regulated pathways or the result of numerous peripheral effects, which could have improved outcomes by indirect mechanisms (Tacer *et al.*, 2010; Bookout *et al.*, 2013). Jiang *et al.* (2018) demonstrated that 14 days of treatment with 1.5 mg/kg rFGF21 (initiated 6 hours postinjury) decreased metabolic dysfunction, neuroinflammation, brain infract size, white matter injury, and improved neurological outcomes after a focal ischemic stroke in 10-week-old diabetic mice. Finally, Restelli *et al.* (2018) demonstrated *in vivo* that increased endoplasmic reticulum (ER) stress in neurons of the brain caused phosphorylation of eukaryotic initiation factor 2 alpha (peIF2α), which in turn stimulated activating transcription factor 4 (ATF4), and subsequently increased neuronal FGF21 expression. Increased hippocampal FGF21 mRNA levels were seen in adult mice with frontotemporal dementia (i.e., P301L Tau mutant mice), and also in tg37 mice inoculated with prions to induce severe neurodegeneration (Restelli *et al.*, 2018). Interestingly, clinically relevant levels of hypothermia potently increased neuronal peIF2α levels *in vitro* (Jackson *et al.*, 2015). Likewise, peIF2α levels are robustly increased in the brain of hibernating squirrels (Frerichs *et al.*, 1998). Thus, the discovery that peIF2α regulates FGF21 expression in the brain reveals a fascinating mechanistic link between the fundamental mechanisms involved in the molecular adaptation to severe hypothermia (i.e., decreased global protein synthesis [GPS] due to increased peIF2α) versus thermogenic singling pathways, which contribute to T_b_ maintenance during mild environmental cold stress (i.e., FGF21). Nevertheless, brain β-klotho was not assessed by Restelli *et al.* (2018), and it is not expected to be present in the hippocampus given the age of animals used in their experiments. Thus, the manner in which increased local FGF21 expression in the adult brain might mediate a direct (paracrine-like) effect is unclear.

FGF21 may improve brain health by indirect mechanisms as well. It stimulates ketogenesis in the liver.^11^ Ketone bodies (acetone, acetoacetate, β-hydroxybutyrate) are efficiently transported into the brain where they serve as an alternative fuel source for oxidative metabolism (Ruderman *et al.*, 1974). Exogenous administration of β-hydroxybutyrate in a rodent model of neonatal HIE decreased neurological injury (Lee *et al.*, 2018a). In the same “Rice-Vannucci” model, Takenouchi *et al.* (2015) showed that hypothermia decreased β-hydroxybutyrate and acetyl-CoA levels in the brain. Similarly, β-hydroxybutyrate levels are slightly decreased in the gray matter in human neonates with HIE during TH, relative to levels after rewarming (Wisnowski *et al.*, 2016). Studies are needed to test if FGF21 supplementation during TH augments ketone substrate availability in the injured brain. Finally, landmark studies by Pawlosky *et al.* (2017) showed that dietary ketone supplementation with an ester of β-hydroxybutyrate for 8 months had remarkable benefits on the brain in 3xTgAD mice (a model of severe Alzheimer's disease). B-hydroxybutyrate supplementation was initiated at 8.5 months of age, which is after the onset of cognitive deficits and neuropathology in these mice (∼6.5 months). B-hydroxybutyrate improved neurological outcome as measured by multiple cognitive tests, decreased pathological β-amyloid and pTau levels, decreased markers of protein/lipid oxidation, and increased levels of N-acetyl aspartate in the hippocampus (Kashiwaya *et al.*, 2013; Pawlosky *et al.*, 2017). FGF21 analogues increased blood β-hydroxybutyrate levels in humans, and thus have potential to target ketogenic neuroprotective mechanisms in the clinic (Gaich *et al.*, 2013).

Blood glucose is another important physiological target of FGF21, and a potential mechanism of its indirect benefits on brain health. Induced hyperglycemia is a complication of TH therapy, which might worsen brain injury outcomes (Cueni-Villoz *et al.*, 2011; Kobata *et al.*, 2017). However, hyperglycemia is managed with trepidation in the neuro-intensive care unit (ICU) because of the risk of exacerbating CNS damage by induced hypoglycemia in the brain with insulin therapy (Forni *et al.*, 2015). FGF21 also decreases blood glucose (Kwon *et al.*, 2015). However, unlike insulin, studies in rodents and primates showed that FGF21 normalizes blood glucose levels without inducing hypoglycemia even at a very high dose (Kharitonenkov *et al.*, 2007) (i.e., there is a ceiling effect by which FGF21 decreases blood glucose no further). Thus, FGF21 might be a far safer drug versus insulin to control glycemia in neurocritically ill patients. Of note, the glucose-lowering actions of FGF21 are not as potent in obese human subjects versus in preclinical animal studies (Gaich *et al.*, 2013). This may relate to species-specific differences in FGF21 signaling, or perhaps result from FGF21 resistance seen in obesity (Markan *et al.*, 2017). Thus, the glucose-lowering action of FGF21 may be more potent in metabolically healthy (younger) humans suffering from acute injuries such as TBI. Furthermore, the contribution of metabolic disturbances in glucose homoeostasis and insulin insensitivity is well recognized in Alzheimer's’ disease, and FGF21 may have the utility to reverse that component of pathogenesis (Yarchoan and Arnold, 2014; Willette *et al.*, 2015; Rodriguez-Rodriguez *et al.*, 2017).

The therapeutic time window is another important factor to consider germane to FGF21's potential for neuroprotection via direct and/or indirect mechanisms. It might be that promoting brain recovery by targeting global improvements in baseline metabolism will have the greatest success in chronic neurodegenerative diseases because therapies can be applied long term, and thus, the benefits of FGF21 would be allowed to evolve over weeks, months, or years. In contrast, in the setting of acute neurocritcal care, the therapeutic time window is comparatively short—particularly if using FGF21 as an adjuvant for TH, which is generally applied in the hospital over 24–72 hours. One might hypothesize that particularly in infants or toddlers, where β-klotho is present, immediate IV injection of FGF21 could serve as a bridge to the induction of hypothermia—in some scenarios such as interhospital transport delaying the application of cooling, or simply augment the use of hypothermia. Thus, the direct neuroprotective effects of FGF21 may be more important in the setting of acute brain injury. Nevertheless, Xu *et al.* (2009) reported that a single FGF21 bolus decreased blood glucose within 1 hour after injection in obese diabetic mice. Thus, FGF21 therapy may prove useful to rapidly target glucose in the neuro-ICU, and possibly to target other peripherally mediated mechanisms, ultimately promoting neuronal survival.

### Irisin

Fibronectin-like III domain containing 5 (FNDC5) is a single-pass transmembrane protein that is predominantly expressed in the muscle (Huh *et al.*, 2012). Seminal work by Bostrom *et al.* (2012) reported that FNDC5 levels were increased twofold in blood plasma in adult humans after endurance exercise, which was mediated by upstream activation of peroxisome proliferator-activated receptor γ coactivator-1α (PGC-1α). Treatment of adipose cells *in vitro* with recombinant full-length FNDC5 activated PPAR-α, and increased UCP1 expression, oxygen consumption, and mitochondrial biogenesis (Bostrom *et al.*, 2012). Finally, proteolytic cleavage at the c-terminus of full-length FNDC5 caused systemic release of a glycosylated protein fragment (irisin) during exercise, which mediated the aforementioned cell signaling changes in adipose cells in mice *in vivo* (Bostrom *et al.*, 2012).

Irisin is increased by cooling. Lee *et al.* (2014) showed that cold shivering in humans significantly increased serum irisin levels. Moreover, stronger shivering responses were positively associated with higher irisin levels among study participants (Lee *et al.*, 2014). Rhythmic muscle contractions occur during both exercise and shivering, and appear to be the link driving increased irisin expression/release. The authors also validated the authenticity of irisin by mass spectrometry; this merits additional discussion later and is expanded on below.

Irisin is also neuroprotective. Li *et al.* (2017a) reported that 200 μg/kg irisin via the tail vein, given 30 minutes after middle cerebral artery occlusion (MCAO) to model stroke, decreased cerebral infarct volume 3 days later. Similarly, Asadi *et al.* (2018) reported that administration of 7.5 μg/kg irisin directly into the brain (intracerebroventricular [ICV]) in MCAO-stroke-injured rats reduced neurological deficits, decreased infarct size, decreased brain edema, and decreased TUNEL staining and other markers of apoptosis. Curiously, BBB damage measured by Evans Blue extravasation was unaffected. In other studies, irisin directly increased brain-derived neurotrophic factor (BDNF) in cortical neurons (Wrann *et al.*, 2013). The Asadi study observed maximum benefit at a relatively high therapeutic dose (7500 ng/kg) (Asadi *et al.*, 2018). For comparison, blood levels in humans after exercise is ∼4.3 ng/mL (Jedrychowski *et al.*, 2015). Nevertheless, germane to its possible endogenous neuroprotective role, studies have detected irisin, using mass spectrometry, in cerebrospinal fluid (CSF) of humans in the range of ∼0.26–1.86 ng/mL, but it has not been confirmed if it crosses the BBB or is produced locally (Ruan *et al.*, 2018). Studies in rats and mice indicate that full-length FNDC5 protein (the precursor of irisin) is abundant in the neonatal developing brain but is almost absent in the adult rodent brain (Tanhaei *et al.*, 2018); these findings parallel work from our group showing that protein levels of neuroprotective CSPs and β-klotho are high in the developing infant brain but absent in adults (Jackson *et al.*, 2018). It remains to be elucidated if elevated brain FNDC5 levels during infancy represent yet another example of the manner in which the young brain is privileged to developmentally regulate neuroprotective cold stress defense mechanisms, which may uniquely communicate some of the benefits of TH at that age. Furthermore, it appears that differential expression of several microRNAs (miRNAs), in an age-dependent manner, is partially responsible for blocking FNDC5 protein translation in the adult brain (Tanhaei *et al.*, 2018). The FNDC5/miRNA regulatory mechanism is yet to be confirmed in humans, but studies show FNDC5 mRNA expression is extremely low in the human adult brain, whereas it is abundant in the muscle as expected (Huh *et al.*, 2012). Thus, these findings support the notion that CSF-irisin may be derived from peripheral sources (at least in adults).

Implementing TH in patients involves careful attention to prevent shivering. The concern is that shivering leads to decreased brain tissue oxygenation due to higher consumption by muscle, and also increases metabolism, which might reverse a key mechanism mediating cooling-induced neuroprotection (Oddo *et al.*, 2010). Thus, sedatives and neuromuscular blockers are routinely used to stabilize patients and control shivering (Choi *et al.*, 2011a). Recent findings on irisin raise important questions relevant to shivering prevention protocols used during temperature management of the neurocritically ill—does sedation/neuromuscular blockade alter or prevent irisin release during TH? Might irisin represent an endogenous beneficial/neuroprotective cell signaling molecule activated by shivering that should be allowed to manifest? If so, is irisin supplementation in sedated patients during or after TH a reasonable alternative? Research is needed to address these and other critical questions, and to determine if they hold promise for novel solutions to better optimize TH, particularly in adults. In addition, irisin administration decreased myocardial and pulmonary injury after ischemia in animal studies (Chen *et al.*, 2017a; Wang *et al.*, 2017). Thus, the benefits of irisin during TH extend beyond the brain, and its upregulation may be particularly advantageous in conditions where there is a risk of multiorgan injury, such as in cardiac arrest patients. Finally, germane to neurodegenerative diseases, muscle wasting is accelerated in Alzheimer's patients and is associated with brain atrophy (Burns *et al.*, 2010). Future studies are needed to explore if irisin signaling is compromised in that population, and if it represents a potential therapeutic target.

We encourage future investigation on irisin in the setting of acute and chronic brain injury, but careful attention must be given to the methodologies used. Irisin levels are most commonly analyzed by enzyme-linked immunosorbent assay (ELISA). The limitations of ELISA, combined with questions surrounding the atypical translational start codon (ATA) of FNDC5 in humans (Raschke *et al.*, 2013), and also other unique methodological challenges relevant to detecting irisin by Western blotting (see reference for more details—Jedrychowski *et al.*, 2015), led to serious contestation on the existence of irisin, amid reports that the commercially available ELISAs detected artifacts and did not measure a bona fide signal (Atherton and Phillips, 2013; Albrecht *et al.*, 2015). In recent years, independent groups have confirmed the existence of irisin, and quantified the levels in human samples using absolute quantification (AQUA) mass spectrometry (Lee *et al.*, 2014; Jedrychowski *et al.*, 2015; Chen *et al.*, 2017a; Ruan *et al.*, 2018). Thus, it is a genuine hormone that is increased in response to exercise and shivering (Lee *et al.*, 2014). Nevertheless, concerns raised by the counter articles merit consideration. For instance, it has been shown by mass spectrometry that FNDC5 antibodies detect both nonspecific targets and bona fide irisin (Lee *et al.*, 2014). Thus, given the controversy, it would be prudent at this investigative stage to use multiple techniques to confirm key findings on irisin levels in patient/subject samples, rather than rely exclusively on ELISAs, which preclude assessment of antibody specificity.

Exploration of irisin in the setting of neurocritical care and in chronic neurodegenerative disease is in its infancy; however, two studies in 2018, the first to our knowledge, have reported on serum irisin levels in humans with brain injury. Decreased irisin levels (i.e., lowest quartile among patients) were associated with worse short-term neurological outcome after ischemic stroke (odds ratio [OR] 1.94; 95% confidence interval [CI] 1.19–3.42), increased mortality (OR 1.66; 95% CI 1.11–3.07), and poststroke depression (OR 1.75; 95% CI 1.15–2.65) (Tu *et al.*, 2018a, 2018b). The results are intriguing, but it is unclear if stroke severity alters irisin levels, if levels directly contributed to outcome, or if irisin is a biomarker of individuals with unfavorable underlying physiology related to preexisting health problems before ischemic brain injury. Most importantly, irisin levels were measured only by ELISA in both studies, and the findings should be confirmed by mass spectrometry.

### Meteorin-like

Peroxisome proliferator-activated receptor γ coactivator-1α4 (PGC-1α4) overexpressing mice have increased muscle strength (hypertrophy), energy expenditure, and decreased WAT depots (Rao *et al.*, 2014; Ruas *et al.*, 2012). Rao *et al.* (2014) identified the hormone Meteorin-like (Metrnl) in a screening assay to detect secreted factors involved in fat mobilization, downstream of PGC-1α4 expression in muscle. They also found that acute environmental cold stress (24 hours/4°C) robustly increased Metrnl mRNA levels selectively in BAT/WAT, and increased protein levels in blood; the specificity of the anti-Metrnl antibody used to confirm changes in protein levels was validated in knockout (KO) mice in these studies (Rao *et al.*, 2014).

Circulating Metrnl activates anti-inflammatory pathways in macrophages. Liver-specific Metrnl overexpressing mice had increased numbers of M2-type (Arg1+) macrophages in adipose tissue, as well as increased expression of anti-inflammatory genes (IL-10, TGF-β, IL-4, and IL-13) (Rao *et al.*, 2014). Furthermore, M2 macrophages secreted norepinephrine (>twofold increase) in the adipose tissue, which in turn stimulated thermogenic mechanisms in WAT. Metrnl-induced conversion of macrophages into an M2 phenotype was blocked by ablation of eosinophils in ΔdbIGATA transgenic mice (Rao *et al.*, 2014).

CNS immune cells, including resident microglia and infiltrating macrophages, alter the extracellular microenvironment after a brain injury (Lan *et al.*, 2017). M1-type (iNOS+) microglia/macrophages release proinflammatory cytokines, whereas M2 cells release anti-inflammatory factors. Truettner *et al.* (2017) demonstrated in rats that TH (4 hours/33°C) increased the ratio of M2:M1 microglia/lymphocytes in the injured cortex after a fluid percussion TBI. Furthermore, TH increased the expression of anti-inflammatory genes, including IL-10 and TGF-β. Because Metrnl causes similar phenotypic changes on peripheral immune cells and is induced by cold stress, future studies are needed to test if Metrnl (1) penetrates the BBB, (2) promotes conversion of M2 microglia in the CNS, and (3) if Metrnl administration might selectively boost the component of neuroprotective cooling, which targets toxic neuroinflammation in the brain.

There are little data on Metrnl signaling in brain, or in cells of the CNS such as neurons. A PubMed search on the terms “Meteorin-like AND Brain” yields three articles. None of these articles directly relate to Metrnl-regulated pathways in the brain. Furthermore, the search terms “Meteorin-like AND Neuroprotection” yield zero articles. To our knowledge, the only available data on the effect of Metrnl in neurons are found in the Supplementary section from the Rao study; the authors validated the activity of a recombinant Metrnl-Fc fusion protein on primary cortical neuron cultures *in vitro*, before testing its effects on signaling mechanisms in the muscle/adipose tissue *in vivo* (Rao *et al.*, 2014). Metrnl-Fc dose dependently increased phosphorylation of signal transducer and activator of transcription-3 (STAT3) in primary neurons (Rao *et al.*, 2014). Choi *et al.* (2011b) showed that hypothermia decreased STAT3 phosphorylation in the brain in a rat model of transient MCAO. This may be an undesirable effect of TH given that (1) increased STAT3 phosphorylation is vital for estradiol-mediated CA1 hippocampal neuroprotection in a model of cerebral global ischemia, and (2) selective inhibition of astrocytic STAT3 in transgenic mice exacerbates white matter damage in a perinatal model of inflammation-mediated brain injury (Nobuta *et al.*, 2012; Sehara *et al.*, 2013). Thus, additional research is needed to test if cold stress-mediated Metrnl secretion might have desirable effects on STAT3 activation in the brain during hypothermia.

### Sex hormone binding globulin

Sex hormone binding globulin (SHBG) is a major protein carrier of androgens and estrogens discovered in the 1960s (Rosenbaum *et al.*, 1966). While not a hormone *per se*, it merits discussion. SHBG binds with highest affinity to dihydrotestosterone (DHT), followed by 2-methoxyestradiol > testosterone > estradiol > methyltrienolone > cortisol (Hryb *et al.*, 1990). Before the 1990s, the prevailing view was that SHBG limited the availability of free sex hormones in the blood (i.e., 1–2% of circulating androgens/estrogens are in the unbound state (Dunn *et al.*, 1981)). This led to the “free hormone hypothesis,” which stipulated that only unbound hormones in plasma have biological activity, and thus, the main purpose of SHBG is to inhibit the effects of sex hormones (Giorgi, 1980; Mendel, 1989). Subsequent studies changed thinking. Hryb *et al.* (1985) showed that SHBG binds to high-affinity orphan receptors located on the membrane surface of prostate cells. Later, they developed a kinetic model to describe the interaction of unbound versus steroid-bound SHBG with its orphan receptor (Hryb *et al.*, 1990); SHBG prebound with hormone failed to attach to surface receptors (regardless of the occupying steroid). In contrast, hormone-free SHBG binds surface receptors with high affinity (receptor-primed SHBG), and on subsequent stimulation with steroids, increased the activation of intracellular cAMP (Hryb *et al.*, 1990). Hammes *et al.* (2005) later elaborated on the upstream signaling mechanisms by showing that receptor-primed SHBG led to the endocytosis of testosterone in rat choriocarcinoma cells. Furthermore, megalin was identified as the SHBG orphan receptor (Hammes *et al.*, 2005). Consistent with the notion that SHBG plays an important role in facilitating steroid activity rather than inhibiting it, megalin KO mice had severe developmental abnormalities in reproductive organs caused by deficiencies in sex hormone signaling (Hammes *et al.*, 2005). Finally, SHBG is endocytosed in neuronal hippocampal HT22 cells *in vitro*, and in cells of the brain *in vivo* (Caldwell *et al.*, 2007).

Two recent studies implicate SHBG as a potential target in neuroprotective cooling (Fig. 4). Deng *et al.* (2018) multiplexed lectin chromatography with mass spectrometry to analyze the glycoproteome in cardiac arrest patients treated with TH, and compared the levels of identified proteins in patients who progressed to a good versus poor neurological outcome. Furthermore, two different lectins were used, concanavilin A (ConA) or wheat germ agglutinin (WGA), which preferentially bind different glycan moieties (i.e., enrich different subsets of glycated proteins). Twenty-three glycoproteins (out of 640) increased in TH-treated patients who had a good neurological outcome. SHBG was among the top 5 (i.e., highest relative levels) in patients with good neurological outcome, and surprisingly was detected by both the ConA and WGA enrichment techniques (Deng *et al.*, 2018). Welinder *et al.* (2016) published the second notable study germane to SHBG and hypothermia. They analyzed the global proteome for the first time in awake versus hibernating subadult brown bears using Q-Exactive mass spectrometry. Subadult bears are roughly comparable with a 6–9-year-old child, based on the relative age of prepuberty. The largest hibernation-dependent change among all blood analytes was an enormous 45-fold increase in the protein levels of glycosylated SHBG (Welinder *et al.*, 2016). Other proteins increased (or decreased) during hibernation, and the pattern of change was remarkably similar to the proteomic profile of adult cardiac arrest patients treated with TH who had a good neurological outcome (Welinder *et al.*, 2016; Deng *et al.*, 2018). The implications of these findings are unclear but raise important questions. Might TH produce the greatest neurological benefit in people (for unknown reasons), manifesting proteomic changes reminiscent of hibernation? Otherwise, could the short list of hibernation responsive proteins serve as a target engagement biomarker panel of neuroprotective TH in patients? Research is needed to address these questions. Also, bears are among the largest hibernating mammals, and T_b_ during hibernation declines to a nadir of ∼30–32°C during winter, which is within the range of mild/moderate TH used for neuroprotection in humans (Toien *et al.*, 2011; Welinder *et al.*, 2016). In contrast, artic squirrels maintain a T_b_ of 0°C or lower during hibernation (Barnes, 1989). Thus, adaptive cold stress mechanisms in hibernating bears may have more in common with hypothermic physiology in humans versus smaller mammals such as squirrels.

There is limited evidence to support a direct neuroprotective role of SHBG. For instance, a PubMed search on the terms “SHBG AND Neuroprotection” yields zero articles. This may be because studies have largely focused on the free hormones that SHBG regulates. A PubMed search on the terms “Testosterone AND Neuroprotection” yields 102 articles, and “Estrogen AND Neuroprotection” yields 906 articles. However, a 2017 subanalysis on data collected over 35 years, as part of The Coronary Artery Risk Development Young Adults Study (CARDIA), found an intriguing association in blood SHBG levels versus brain volume (magnetic resonance imaging [MRI]) in middle-aged men (Elbejjani *et al.*, 2017). Specifically, higher SHBG levels correlated with increased total white matter in brain. The association was largest in temporal and frontal lobe white matter (Elbejjani *et al.*, 2017). In contrast, higher SHBG levels correlated with decreased gray matter but only in the parietal lobe (Elbejjani *et al.*, 2017). The consequence of SHBG levels on individual differences in gray versus white matter volume and on brain function merits additional study—particularly if SHBG is increased by TH and might influence white matter recovery.

## Induction of CSPs During Cold Stress: Potential Applications for Neuroprotection

**FIG. 5.**
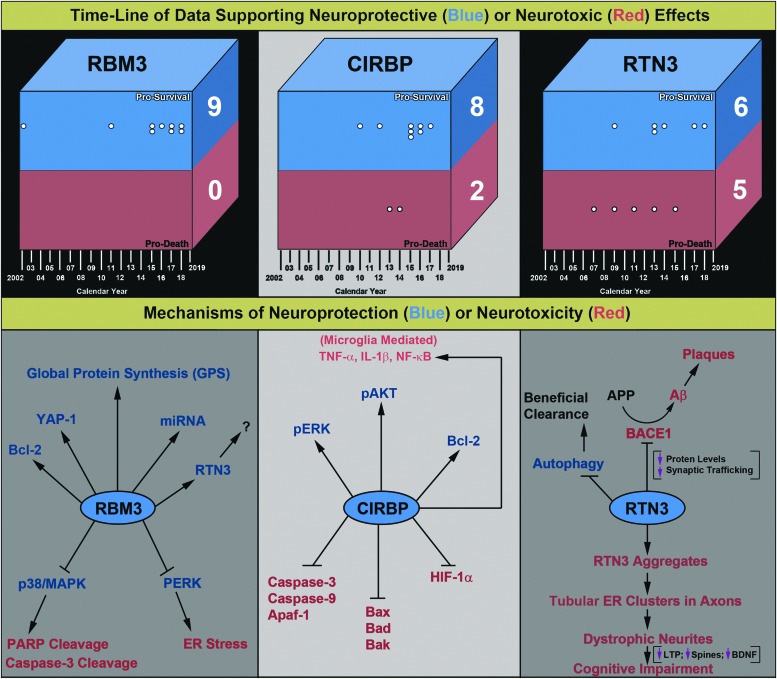
Evidence supporting either a direct neuroprotective or neurotoxic function of RBM3, CIRBP, or RTN3 in the brain. Literature on RBM3, CIRBP, and RTN3 was obtained via PubMed. All articles were screened via an initial abstract review. A secondary search via Google was done to identify any additional articles not referenced in PubMed. Studies using *in vitro* neuronal injury paradigms (cell lines or primary neurons) or *in vivo* brain injury/disease models were analyzed in-depth (i.e., if available). One article was in Chinese and converted to English using Google translate. Purely observational studies were identified and excluded. This figure incorporates studies that (1) overexpressed, (2) knocked down, or (3) incubated neuronal cells/tissues with a recombinant CSP (or any combination of the three) *in vitro* and/or *in vivo*, to generate a direct conclusion germane to a given CSP protective versus detrimental function(s). For the purpose of this review, we did not rate the “quality of the evidence” but noted that the scientific rigor varied considerably across studies. *Top*: findings were organized into “Battlefield Boxes,” which summarize the opposing sides of evidence that support either neuroprotective (*blue region*) or neurotoxic (*red region*) roles of CSPs. *White dots* indicate individual studies and each is aligned with the year of publication. *Stacked dots* indicate multiple studies published in the same year. A total “Score” was given (*large white numbers* on the *right side* of the *squares*), which is the sum of all studies that supported either protective or detrimental functions of each CSP. The literature epoch spans approximately two decades from 2002 to 2019. *Bottom*: an overview of the diverse cell signaling mechanisms reported to mediate neuroprotective versus neurotoxic effects of CSPs. The proposed mechanisms are based on the experimental data presented by articles shown in the *Battlefield Boxes*. All articles are cited in the main text, and also listed here in order of publication date. *RBM3:* Kita *et al.*, Chip *et al.*, Zhu *et al.*, Peretti *et al.*, Yang *et al.*, Bastide *et al.*, Zhuang *et al.*, Yang *et al.*, Xia *et al.* CIRBP: Saito *et al.*, Li *et al.*, Rajayer *et al.*, Zhou *et al.*, Liu *et al.*, Zhang *et al.*, Li *et al.*, Zhang *et al.*, Wang *et al.*, Chen *et al.* RTN3: Hu *et al.*, Shi *et al.*, Shi *et al.*, Chen *et al.*, Shi *et al.*, Teng and Tang, Araki *et al.*, Shi *et al.*, Sharoar *et al.*, Bastide *et al.*, Zou *et al.* CIRBP, Cold inducible RNA binding protein; RTN3, reticulin-3.

CSPs increase during cold stress and mediate cold adaptation in cells. They are usually retained intracellularly (i.e., not secreted), and levels progressively increase as core T_b_ falls below thermoneutrality. Whereas CSHs are integral to cold defenses, CSPs are integral to cold tolerance and are potently recruited once adaptive thermogenesis mechanisms fail to maintain normothermia; however, activation of CSH versus CSP mechanisms overlaps to some extent. Cold-induced CSP expression appears to be intrinsic to most mammalian cell types (at least *in vitro*). Thus, CSP molecules have the capacity to affect signaling pathways in potentially any organ subjected to extended periods of hypothermia, which may be an important aspect of their benefit in the setting of total body cooling for neuroprotection. Recent studies, showing that overexpression of CSPs in the hippocampus mediates incredible and enduring histological and behavioral improvements in normothermic mice afflicted with severe neurodegenerative diseases, have intensified interest in cold-regulated cell signaling mechanisms in the brain. Here we review the three mammalian CSPs with the most clinical interest to date (Fig. 5).

### RNA binding motif 3

Danno *et al.* (2000) were the first to show that cooling (32°C for 24 hours) increases RBM3 levels in mammalian cells. Subsequent studies confirmed that hypothermia increases RBM3 levels in primary neurons and in brain tissue, but the majority of evidence comes from *in vitro* and *in vivo* experiments that used models most relevant to the developing CNS. Chip *et al.* (2011) observed increased RBM3 mRNA levels in brain cortical organotypic slice cultures (COSCs) incubated at 32°C for 72 hours. Notably, RBM3 induction postcooling was blunted almost threefold in COSCs prepared from PND21 pups (∼toddlers) versus cortices from PND4 neonates (∼infants) (Chip *et al.*, 2011). Furthermore, baseline RBM3 expression at 37°C was decreased in PND21 versus PND4 cortices (Chip *et al.*, 2011). Thus, slight differences in postnatal age (by just a few weeks) profoundly altered the magnitude of RBM3 gene expression after cooling in the intact mouse brain *ex vivo*. This is a clear example as to why the Responsivity of Cold Stress Pathways is an important concept to consider germane to the efficacy of cooling.

Similarly, we reported that 33°C for 48 hours robustly increased RBM3 protein levels in cultured immature DIV6 primary rat cortical neurons but not in mature DIV26 neurons (Jackson *et al.*, 2015). Cooling also increased RBM3 levels in pure primary rat astrocyte monocultures (Jackson *et al.*, 2015). Larrayoz *et al.* (2016) reported that an environmental temperature of 8°C increased RBM3 protein levels in the rat eye; however, the magnitude of induction was greater in neonates versus adults. In neonates, RBM3 increased ∼30–40% above control levels 24 hours after exposure to 8°C for 15 minutes. In adults, RBM3 increased ∼10% above control levels 24 hours after exposure to 8°C for 3 hours (Larrayoz *et al.*, 2016). Thus, a 12 × shorter cold stress period induced a 4 × greater RBM3 response in neonates. Of note, at normal ambient temperature (24°C), baseline T_b_ in neonates was 31°C, whereas in adults T_b_ was maintained at 37°C (Larrayoz *et al.*, 2016). Also, 15 minutes at 8°C decreased core T_b_ by 10°C in neonates (i.e., nadir of 21°C). In contrast, 3 hours at 8°C decreased core T_b_ in adults by 3°C (i.e., nadir of 34°C) (Larrayoz *et al.*, 2016). Newborns have a large surface area relative to volume, which makes them highly susceptible to hypothermia. Also, the large (uninsulated) head relative to body mass is a major source of heat loss (Karlsson, 1996). Thermoneutrality in a naked baby is achieved at an environmental temperature of ∼32°C, whereas it ranges from 25°C to 28°C depending on age, weight, and levels of clothing (e.g., fully clothed in a cot) (Hey and O'Connell, 1970). Thus, heightened responsivity of CSP pathways in neonates may serve as a compensatory mechanism to combat cold stress, given the limited utility of nonshivering thermogenesis mechanisms to offset heat loss.

Time course studies on CSP expression profiles in normothermic brains of rats, mice, and in humans agree that RBM3 protein levels are highest in neonates/infants, rapidly decrease with increasing age, and are low or absent in adults (Pilotte *et al.*, 2009; Chip *et al.*, 2011; Jackson *et al.*, 2018). Xia *et al.* (2018) reported that RBM3 KO mice have abnormal brain development and dysfunctional neuronal differentiation only after an exposure to cold stress *in utero*. Thus, increased baseline RBM3 levels in the fetal brain may have evolved to protect neurodevelopmental processes against deleterious decreases in maternal temperature, such as due to sudden exposure to extreme weather conditions or periods of starvation (associated with hypothermia), which frequently impact mammals and likely were a common threat to early human ancestors. Interestingly, intrauterine hypoxia in pregnant mice decreased RBM3 mRNA levels in the unborn fetal brain (Trollmann *et al.*, 2010). More studies are needed to determine if birth asphyxia induces long-term impairments to RBM3 cold adaptive mechanisms in surviving newborns, and what effect it might have on neurodevelopmental outcomes. It is important to note that, in the Xia study, cold stress was induced in pregnant mice by intraperitoneal (IP) 5-AMP bolus injection (700 mg/kg), followed by housing animals in a temperature-controlled chamber (5–10°C) for 24 hours (Xia *et al.*, 2018). The lethal dose (LD50) of 5-AMP in mice is 830 mg/kg (IV) and 4000 mg/kg (IP). At sublethal concentrations it induces a profound hypometabolic state characterized by hypothermia, decreased glycolysis, decreased oxidative phosphorylation, decreased oxygen delivery, decreased heart rate, and decreased blood pressure (Daniels *et al.*, 2010; Zhang *et al.*, 2013). These physiological changes are not benign. A 2000 mg/kg (IP) 5-AMP worsened histological/neurological outcomes and increased mortality in stroke-injured mice, despite inducing clinically relevant levels of hypothermia (Zhang *et al.*, 2013). Severe hypotension induced by 5-AMP may have augmented ischemia and contributed to the exacerbation of acute brain injury. Thus, the combination of 5-AMP plus environmental cooling, often referred to as a “hibernation-like” protocol, is distinct from classic “hypothermia” and does not necessarily reflect the normal sequelae of molecular events induced by TH in patients, to treat acute brain injury. Regardless, 5-AMP hibernation-like cooling may yield valuable molecular insights into the mechanisms of RBM3 induction and lead to better therapies targeting this pathway in the brain. Related to that point, Peretti *et al.* (2015) showed for the first time in a nonhibernating species that cooling mice to 16°C (via 5-AMP injection plus external cooling) increased RBM3 protein levels in the adult brain. Additional findings of their study are discussed later findings that have not been reported using hypothermia alone.

The depth of hypothermia also influences RBM3 expression. Tong *et al.* (2013) compared the effect of mild (33.5°C) versus deep (17°C) hypothermia on CSP expression in organotypic hippocampal slice cultures (OHSCs) prepared from P5 mouse pups. Cooling for 24 hours to 33.5°C potently increased RBM3 mRNA and protein expression, whereas cooling to 17°C did not. Likewise, recent characterization of RBM3 in the Pashmina goat revealed that moderate hypothermia to 30°C increased RBM3 mRNA levels, whereas deep hypothermia to 15°C did not (Zargar *et al.*, 2015). Studies from our group found that hippocampal RBM3 levels in adult rats were not increased (i.e., below detection limits) immediately following 75 minutes of DHCA to 16°C, and were also not increased 24 hours postresuscitation (presented abstract) (Drabek *et al.*, 2017). Together the results suggest that deep hypothermic temperatures may preclude the induction of protective RBM3 mechanisms. However, as discussed above, 5-AMP-induced cooling uniquely appears to augment RBM3 at 16°C (Peretti *et al.*, 2015) and merits additional investigation. Such an approach could have clinical utility in the setting of DHCA to promote neuroprotection during prolonged surgeries, necessitating a period of controlled ischemia, and in which patients are already on advanced life support thus reducing concerns related to managing the sequelae of 5-AMP overdose. Finally, although deep hypothermia is highly protective during ischemic insults, it is generally not effective when used after acute brain injury. One hour of mild or moderate hypothermia (34°C or 30°C, respectively) immediately after VF cardiac arrest in dogs improved neurological outcomes, whereas deep hypothermia (15°C) worsened outcomes (Weinrauch *et al.*, 1992). It is unclear if RBM3-mediated survival mechanisms in the brain (or periphery) could have mediated some of the benefits of mild/moderate TH after cardiac arrest in these studies but were abrogated with deep cooling. Additional study is warranted.

RBM3 is a potent neuroprotectant. Kita *et al.* (2002) used high-throughput real-time polymerase chain reaction (RT-PCR) to identify genes affected by Huntington's disease (HD) neuropathology in genetically modified rat neuronal PC12 cells. RBM3 mRNA decreased in cells expressing a toxic polyglutamine fragment (HD-74Q). Furthermore, exogenous RBM3 overexpression in human neuronal SK-N-SH cells inhibited HD-74Q-mediated cell death versus an empty vector control (Kita *et al.*, 2002). To our knowledge, this is the only report on RBM3 in the setting of HD, and also happens to be the first study to show that RBM3 is neuroprotective. Recent work suggests that the therapeutic utility of RBM3 in HD merits further consideration. Mao *et al.* (2016) reported that overexpression of HD-104-Q in primary mouse cortical neurons induced a new type of necrosis (distinct from RIPK pathways), termed ballooning cell death. The upstream mechanisms involved inhibition of the transcription factor yes-associated protein (YAP), which normally promotes survival signaling. Specifically, aggregates of mutant Huntington sequestered YAP and prevented its nuclear activity; overexpression or KO of YAP inhibited or exacerbated HD-104-Q neuronal death, respectively (Mao *et al.*, 2016). Mueller *et al.* (2018) recently confirmed that nuclear YAP localization is downregulated in cortical neurons of HD patients. As mentioned above, RBM3 KO mice exposed to cold stress *in utero* have neurodevelopmental abnormalities. The mechanism involves (loss of) RBM3 induction of the YAP (Xia *et al.*, 2018). YAP levels are robustly increased in fetal brain after cold stress in wild type but not in RBM3 Kos (Xia *et al.*, 2018). Furthermore, the 3’UTR of the YAP gene has seven RBM3 recognition sites, and binding to these cis-acting elements increases YAP mRNA half-life (Xia *et al.*, 2018). Thus, evidence suggests that RBM3 targets crucial cell signaling pathways uniquely involved in the etiology of HD.

Chip *et al.* (2011) showed that pretreatment with hypothermia (32°C) for 24 hours decreased PARP cleavage, DNA fragmentation, as well as LDH release in neuronal PC12 cells injured by staurosporine (i.e., an inducer of caspase-mediated cell death). Blocking RBM3 expression by RNA interference (RNAi) prevented the beneficial effects of hypothermia. Peretti *et al.* (2015) showed that hibernation-like cooling increased RBM3 levels in the brain of adult mice. Blocking RBM3 upregulation by RNAi prevented neuroprotective and cognitive improvements induced by hibernation-like cooling in transgenic mice afflicted with Alzheimer's disease (5XFAD) or inoculated with prions to induce severe neurodegeneration (tg37 mice) (Peretti *et al.*, 2015). Remarkably, exogenous brain RBM3 overexpression in normothermic mice replicated the benefits of cooling in both disease models (Peretti *et al.*, 2015). Zhu *et al.* (2016) demonstrated that *ex vivo* hippocampi (OHSCs) prepared from PND3 RBM3 KO pups had increased neuronal death 24 hours after thapsigargin (SERCA inhibitor) treatment to induce ER stress. Interestingly, thapsigargin-mediated neuronal death, involving PERK/CHOP activation and downregulation of Bcl-2, was exacerbated in Kos at 37°C but not at 32°C (Zhu *et al.*, 2016). Infants uniquely express high levels of RBM3 in the normothermic hippocampus (Jackson *et al.*, 2018). Thus, future studies are needed to test if the injured normothermic newborn brain might be more resilient to ER stress-mediated cell death mechanisms relative to adults due to higher baseline RBM3 levels. Moreover, Zhu *et al.* (2016) also observed that RBM3 Kos had increased hippocampal peIF2α levels at baseline and during cold stress. Increased peIF2α stimulates FGF21 gene expression in the hippocampus (Restelli *et al.*, 2018). Moreover, we found that recombinant FGF21 increased RBM3 levels at 36°C in cortical neurons *in vitro* (Jackson *et al.*, 2015). Thus, we speculate that FGF21 levels might be increased in RBM3 KO mice (i.e., a feedback mechanism).

Yang *et al.* (2017) showed that RBM3 overexpression in neuronal SHSY5Ys decreased nitric oxide (NO)-induced cell death. The mechanism of neuroprotection involved inhibition of p38 kinase, and was blocked by simultaneous overexpression of mir-143. Similarly, Zhuang *et al.* (2017) showed that RBM3 overexpression in SHSY5Ys decreased p38 activation, PARP cleavage, and caspase-3 activation, and increased cell viability following ultraviolet radiation injury. Bastide *et al.* (2017) showed in a neurodegenerative model of prion disease that the neuroprotective effects of RBM3 in the hippocampus *in vivo* are mediated, in part, by the expression of reticulin-3 (RTN3). Yang *et al.* (2018) showed that RBM3 overexpression in neuronal SHSY5Ys decreased PARP cleavage and caspase3/7 activity after 24 hours 3 mM MPP+ to model mechanisms of injury in Parkinson's disease. Finally, RBM3 may mediate neuroprotection by additional mechanisms as well. Acute brain injury is well-known to decrease GPS, which precedes the onset of delayed neuronal death (Neumar *et al.*, 1998; de la Vega *et al.*, 2001). RBM3 increases GPS *in vitro* and in the brain *in vivo* by a mechanism that involves upregulation of miRNAs (Dresios *et al.*, 2005; Pilotte *et al.*, 2011; Peretti *et al.*, 2015). Preserving GPS likely promotes neuronal survival.

Although no detrimental effects of RBM3 overexpression have been reported in models of neuronal injury, there may be risks associated with pharmacologically increasing RBM3 systemically. Wong *et al.* (2016) reported that blood RBM3 mRNA levels decreased in febrile children (independent of the underlying illness) versus healthy controls or children with infections who did not have a fever. Thus, increased temperature, not infection or inflammation, mediated decreased RBM3 levels. Furthermore, RBM3 protein levels decreased sixfold in macrophages exposed to hyperthermia (40°C) for 24 hours, in turn mediating an increase in expression of “thermomiRs,” temperature sensitive miRNAs, and in turn downregulated pyrogenic genes (Wong *et al.*, 2016). Thus, lowering RBM3 below baseline levels switches off fever-inducing pathways. The net implications or concern, germane to brain injury, is that in theory pharmacologically increasing RBM3 levels in the periphery for an extended period might increase circulating pyrogens and increase susceptibility to an acquired fever. Such a phenomenon (if confirmed) could be managed effectively in the neuro-ICU as cooling devices are routinely used to clamp temperature in patients with acute brain injury (i.e., TH or TTM). However, it might be problematic in normothermic patients with chronic neurodegenerative diseases, particularly given that even mild hyperthermia (1–2°C) synergizes with CNS damage to devastating effect in models of brain injury (Baena *et al.*, 1997; Sakurai *et al.*, 2012).

There are few options (small molecules or biologics) to therapeutically increase RBM3 levels in neurons. Our group reported (2015) that FGF21 and melatonin augmented RBM3 protein levels in young primary cortical neurons cooled to 36°C for 24 hours but not in cells maintained to 37°C, or in older cultures (Jackson *et al.*, 2015). FGF21 may be useful to target RBM3 in infants/toddlers because β-klotho expression is abundant in the brain at those ages (Jackson *et al.*, 2018). Papadima *et al.* (2017) showed *in vitro* that lithium (1 mM) for a week increased RBM3 mRNA aprpoximately 30–40% above controls in human neural progenitor cells. Toxic compounds also increase RBM3 mRNA levels. Ryan *et al.* (2005) administered domoic acid to activate kainate receptor-mediated excitotoxicity in the mouse brain, and observed a transient increase in RBM3 mRNA 60 minutes postinjection. Baghdoyan *et al.* (2000) showed that RBM3 mRNA increased in human primary CD34^+^ macrophages *in vitro* after 8 hours of treatment with granulocyte/macrophage colony stimulating factor (GM-CSF). Finally, some compounds decrease RBM3 levels. Jo *et al.* (2012) reported that antifreeze protein III improved viability of vitrified mouse oocytes, and subsequently decreased RBM3 mRNA levels on rewarming (presumably caused by reduced cold stress). Laustriat *et al.* (2015) observed an approximately threefold decrease in RBM3 protein levels in human mesodermal precursor cells treated *in vitro* with metformin for 48 hours. Thus, more research is needed to elucidate drugs that target the RBM3 pathway in the brain.

### Cold inducible RNA binding protein

Nishiyama *et al.* (1997) were the first to report that cold inducible RNA binding protein (CIRBP) is a mammalian CSP. Cooling (32°C) increased CIRBP levels within 1 hour in mouse fibroblasts, and highest levels were achieved by 12–24 hours. They also identified diurnal changes in CIRBP mRNA expression in the mouse brain; levels peaked at 6 p.m. and decreased to a nadir at 3 a.m. (Nishiyama *et al.*, 1998). Furthermore, we reported that CIRBP is abundant in the normothermic human infant hippocampus and cortex but is absent in adolescents and in adults (Jackson *et al.*, 2018). Thus, developmental age and circadian rhythm regulate baseline levels of CIRBP in the brain. More work is needed to test if CSHs regulate developmental and/or diurnal expression of CIRBP mRNA in the CNS. However, FGF21 or melatonin, compounds that are both endogenously regulated by circadian cycles *in vivo*, failed to augment CIRBP levels at 36°C in young DIV6 cortical neurons *in vitro* (Jackson *et al.*, 2015).

Subsequent studies confirmed that hypothermia increases CIRBP levels in primary neurons *in vitro* and in the brain *in vivo*. Li *et al.* (2012) reported that exposure of cultured rat cortical neurons to 32°C for 2 hours increased CIRBP levels. Similarly, Zhang *et al.* (2015) reported that exposure of DIV7 cultured rat cortical neurons to 32°C for 12 hours increased CIRBP levels. In contrast, Tong *et al.* (2013) reported that exposure to 33.5°C failed to increase CIRBP levels 4, 24, or 48 hours in *ex vivo* PND5-derived mouse hippocampi, mouse neuronal HT22 cells, or in microglial BV-2 cells. However, CIRBP mRNA levels increased postcooling at all three time points in all three culture systems (Tong *et al.*, 2013). Thus, rapid and sustained increases in CIRBP mRNA levels at 33.5°C did not translate to increased protein levels. We reported similar findings. Cooling to 33°C for 24–48 hours failed to increase CIRBP levels in DIV10-11 rat cortical neuron cultures (Jackson *et al.*, 2015). Additional work is needed to test if a 1°C difference in temperature used across studies (i.e., 32°C vs. 33°C) could have affected CIRBP expression levels. Clarification of the temperature(s) and exposure time(s) required to increase neuronal CIRBP (and other CSPs) may impact study design of future *in vivo* investigations (Chandrasekaran *et al.*, 2015). However, deep hypothermia to 15–17°C failed to increase mRNA or protein levels in fibroblasts, HT-22, BV-2 cells, or *ex vivo* hippocampi obtained from PND5 mouse pups (Nishiyama *et al.*, 1997; Tong *et al.*, 2013). Thus, it appears that the optimal depth of hypothermia to induce CIRBP falls within the mild/moderate range (much like RBM3).

Total body cooling also increases brain CIRBP expression *in vivo*. Wang *et al.* (2016) induced hypothermia (31°C) in adult ∼3-month-old rats under anesthesia for 48 hours, and observed increased CIRBP levels in the hypothalamus at 24–48 hours postcooling. Wu *et al.* (2006) induced TH (∼32–34°C) for 6 hours immediately after ROSC in a rat model of VF cardiac arrest, and observed increased CIRBP levels in the hippocampus. Of note, normothermic VF cardiac arrest-injured controls also had increased hippocampal CIRBP levels versus shams, and cooling augmented the increased expression. The effect of increased brain CIRBP levels on histological and neurological outcomes was not elucidated. Kaneko and Kibayashi (2012) housed adolescent (∼PND42) conscious mice in a temperature-controlled chamber set to ∼4°C for 24–48 hours. Rectal temperature (T_b_) decreased to ∼33–35°C 24 hours later, and CIRBP mRNA increased in the olfactory bulb and in the hypothalamus (Kaneko and Kibayashi, 2012). Similarly, Wang *et al.* (2015) observed increased CIRBP levels in the hippocampus, cortex, cerebellum, heart, muscle, liver, and BAT in adolescent (∼PND42) conscious rats housed at 4°C for 6 hours/d, for a total of 14 consecutive days. On experimental day 1 (PND42), 6 hours of cooling decreased rectal temperature to ∼32°C. Each consecutive day thereafter, the decrease in T_b_ was progressively smaller and absent on day 13 (PND55) (Wang *et al.*, 2015). It remains to be determined if chronic environmental cold stress similarly increases CIRBP levels in the brain of adult rodents, and in which thermogenesis mechanisms have fully matured.

Studies suggest that CIRBP is neuroprotective. However, evidence is limited. Saito *et al.* (2010) reported that CIRBP knockdown in MEB5 mouse neural stem cells prevented hypothermia-mediated neuroprotection (32°C/24–48 hours) induced by growth factor deprivation, and increased the number of apoptotic nuclei measured by Hoechst33342 staining. Li *et al.* (2012) observed an approximately twofold increase in CIRBP levels in primary rat cortical neurons after brief cooling (2 hours to 32°C). Hypothermia prevented increased apoptotic Annexin/PI cell labeling (quantified by flow cytometry) and decreased caspase-3 activation 2 hours after treatment with 100 μM H_2_O_2_, whereas CIRBP knockdown blocked cooling-induced neuroprotection (Li *et al.*, 2012). Liu *et al.* (2015) treated mouse Neuro2a cells with 100 ng/mL recombinant CIRBP at 37°C and after 60 μM H_2_O_2_ injury, survival was increased (∼15–20%) in cells treated versus controls. Treatment also increased phosphorylation (activation) of the protective kinases pERK and pAKT.

Neuronal culture age alters the expression of cell death proteins *in vitro*, and naive immature (young) neurons have the highest level of proapoptotic caspase-3 (Lesuisse and Martin, 2002; Kim *et al.*, 2007). Zhang *et al.* (2015) quantified Annexin/PI staining by flow cytometry in uninjured primary rat cortical neuron cultures at DIV4, 7, and 10. DIV7 cultures maintained to 37°C had the largest fraction of PI-stained neurons (late apoptosis) relative to other culture ages, and hypothermia (12 hours to 32°C) decreased PI staining by ∼25%. Furthermore, hypothermia increased protective proteins, including CIRBP, Bcl-2, and AKT—whereas it decreased apoptotic proteins, including Bax, Bad, Bak, caspase-3, caspase-9, and Apaf1 (Zhang *et al.*, 2015). The beneficial effects of cooling were blocked by CIRBP knockdown (Zhang *et al.*, 2015). Li *et al.* (2015) reported similar findings. Hypothermia (32°C) decreased baseline levels of developmentally regulated apoptosis in primary rat hippocampal neurons *in vitro*, and CIRBP knockdown blocked the prosurvival effects of cooling.

CIRBP is also neuroprotective after a TBI. Wang *et al.* (2016) maintained hypothermia (31°C) for 48 hours in anesthetized adult rats administered food and water via gastrostomy. CIRBP levels increased in the hypothalamus within 6 hours of cooling and remained elevated 72 hours later. Furthermore, hypothermia decreased TUNEL staining (apoptosis) in the cortex, hippocampus, and hypothalamus 96 hours after a lateral fluid percussion TBI (Wang *et al.*, 2016). Intrathecal injection of an adenoviral vector to knockdown CIRBP (before injury) decreased target protein levels after hypothermia, and worsened brain injury as indicated by increased TUNEL staining post-TBI (Wang *et al.*, 2016). The lack of traditional preclinical TBI outcome measures prevented a direct comparison of CIRBP protective effects relative to other reports on neuroprotective strategies in this model. Nevertheless, the 48-hour hypothermia protocol is intriguing. In rodents, neuroprotection is achieved with short durations of cooling (e.g., 4 hours to 33°C in fluid percussion TBI) (Truettner *et al.*, 2017). Thus, longer durations of hypothermia are uncommon. It would be interesting to test if other CSPs (e.g., RBM3) increased in the adult rat brain after an extended period of hypothermia.

Finally, CIRBP inhibits chronic hypoxia-induced neuronal death. Zhang *et al.* (2017) overexpressed CIRBP in a mouse cerebellum-derived neural progenitor cell line (C17.2). Hypoxia (1% O_2_/37°C for 24 hours) decreased proliferation (EdU staining) in cells transfected with an empty vector control plasmid but not in cells overexpressing CIRBP. Chen *et al.* (2017b) observed increased protein levels of HIF-1α, Bax, and cleaved caspase-3 in neuronal SHSY5Ys subjected to hypoxia for 48 hours (1% O_2_/37°C). Apoptosis also increased 48 hours later as determined by Annexin/PI staining. CIRBP overexpression prevented the upregulation of prodeath protein targets, including HIF-1α and decreased apoptosis.

However, CIRBP also has neurotoxic effects. Rajayer *et al.* (2013) observed increased brain CIRBP mRNA and protein levels 10 hours after alcohol intoxication in mice. Pro-inflammatory cytokines (TNF-α and IL-1β) increased in the brain in wild-type mice but not in CIRBP Kos. Furthermore, CIRBP, TNF-α, and IL-1β mRNA levels increased in microglial BV-2 cells treated with alcohol for 48 hours, and high levels of CIRBP were detected in the culture medium suggesting increased secretion (Rajayer *et al.*, 2013). Zhou *et al.* (2014) reported that cerebral infarct volume (TTC staining) decreased approximately threefold in CIRBP KO versus Wt mice 30 hours after permanent MCAO to model stroke. Treating BV-2 cells with 1 μg/mL rCIRBP for 20 hours increased TNF-α protein levels in the culture medium, and increased NF-κB gene promoter activity on a reporter assay (Zhou *et al.*, 2014). In contrast, 1 μg/mL rCIRBP failed to significantly increase caspase activity in differentiated human neuronal SHSY5Ys versus untreated controls. However, in the same experiment, treatment with 5 ng/mL rTNF-α significantly increased caspase activity (Zhou *et al.*, 2014). Thus, CIRBP does not appear to be directly toxic to neurons but rather promotes neuroinflammation (in the presence of microglia), which can indirectly augment neuronal injury—although both detrimental and beneficial effects of neuroinflammation are well described (Simon *et al.*, 2017). We have found that treating mixed astrocyte/cortical neuron cocultures with 1 μg/mL rCIRBP immediately before a 75% mechanical stretch-injury, to model *in vitro* TBI, did not exacerbate (or improve) cell death 24 hours later, as measured by increased LDH release (unpublished observations).

Given these conflicting results, more work is needed to clarify the role of CIRBP on neuronal survival and neuroinflammation *in vivo*, particularly in the setting of TH. Indeed, TH has been shown in some studies to inhibit neuroinflammation *in vivo*, including downregulating TNF-α and IL-1β, which has been suggested to be an important mechanism mediating its protective effects on the brain (Meybohm *et al.*, 2010; Tomura *et al.*, 2012). However, as discussed previously in this review, the effects of TH on either neuroinflammation or systemic inflammation are conflicting and inhibitory effects are not always seen clinically (Buttram *et al.*, 2007; Callaway *et al.*, 2008). In addition, to the best of our knowledge, CIRBP has not been directly studied in chronic conditions such as Alzheimer's disease. However, Akila Parvathy Dharshini *et al.* (2018) identified CIRBP among a short list of genes harboring a nonsynonymous substitution selectively in the temporal lobe of Alzheimer's patients, but the implication of that finding remains to be determined. The role of CIRBP in both acute and chronic CNS insults remains to be elucidated.

There are several promising therapies to target CIRBP *in vivo*. Coderch *et al.* (2017) identified the first small-molecule CIRBP inducers. A single IP bolus of compound Zr17-2 (∼6.5 μg/kg) increased CIRBP levels in the lung and in the pancreas 4 days later in normothermic rats and CIRBP levels modestly increased in the cerebral cortex. The mechanism(s) by which Zr17-2 upregulates CIRBP are unclear but may involve stabilization of flexible regions located at the terminal ends of the protein. It is also unclear if Zr17-2 binding alters the functionality of CIRBP (i.e., increases or decreases target engagement). Nevertheless, Zr17-2 is a first step toward the development of drugs that target increased CSPs at 37°C.

Dietary modification is also a potential approach to increase CIRBP levels in the brain. Oishi *et al.* (2013) showed that a 2-week ketogenic diet decreased the average daily T_b_ in mice by 1°C (i.e., within the range of UMH). Furthermore, T_b_ fluctuates in a diurnal pattern, and mice maintained on a ketogenic diet had the lowest absolute temperature late night (nadir to ∼35°C) versus controls, and CIRBP/FGF21 mRNA levels increased in the liver at that time point (Oishi *et al.*, 2013). Total calorie content was equivalent in mice fed a ketogenic versus standard diet, indicating that the induction of UMH, and increased CIRBP expression, was not due to caloric restriction. Exogenous administration of ketones, in some models, improves histological outcomes after an acute brain injury and decreases neuropathology/cognitive impairment in chronic neurodegenerative disease (Kashiwaya *et al.*, 2013; Pawlosky *et al.*, 2017; Lee *et al.*, 2018a). It is unclear if neuronal CIRBP levels might be increased by administration of ketones in the setting of brain injury, and if it influences recovery (i.e., direct CIRBP-mediated neuroprotective effects versus harmful activation of proinflammatory microglia).

### Reticulon-3

The RTN3 gene was identified by Moreira *et al.* (1999). The canonical sequence encodes a ∼25 kDa protein (RTN3A1), and mRNA levels are highest in the brain. Di Scala *et al.* (2005) later reported additional RTN3 splice variants. Moreover, developmental age altered the protein levels of several isoforms in the cerebellum (E18-PND35). The smallest variant (RTN3C) migrated as a double band at ∼16/19 kDa on SDS-PAGE (Di Scala *et al.*, 2005). The 16 kDa protein decreased, whereas the 19 kDa protein increased in PND35 mice. In contrast, canonical RTN3A1 levels were not altered by age (E18-PND35) in the cerebellum (Di Scala *et al.*, 2005). Shi *et al.* (2009a) reported that RTN3A1 is stably expressed in the cerebellum in adult mice (2–24 months); however, levels increased in the hippocampus in an age-dependent manner. Conversely, Kumamaru *et al.* (2004) observed decreased RTN3A1 staining in the adult versus embryonic mouse retina. We also reported decreased levels of RTN3A1/RTN3C in the hippocampus/cortex in adults versus infants (Jackson *et al.*, 2018). Thus, additional work is needed to clarify the levels of RTN3 protein variants in the brain across the life span in rodents and humans.

Bastide *et al.* (2017) reported that RTN3A1 is a novel CSP. Cooling 24 hours to 32°C increased RTN3A1 levels in neuronal SHSY5Ys. Moreover, hibernation-like cooling to 16°C increased RTN3A1 levels in an RBM3-dependent manner in the adult mouse hippocampus (Bastide *et al.*, 2017). Also, hibernation-like cooling decreased neuropathology in mice with prion disease, but the beneficial effects of hypothermia were blocked by RTN3A1 knockdown in the hippocampus (Bastide *et al.*, 2017). Conversely, Chen *et al.* (2011b) reported that RTN3A1 knockdown increased survival (rather than exacerbate damage) in an *in vitro* model of prion disease in neuronal N2a cells maintained to 37°C. The mechanism of protection involved the activation of autophagy, which increased prion clearance. Finally, in the setting of apoptosis-inducing stimuli, RTN3A1 overexpression decreased cell death after serum deprivation or chemical injury with staurosporine or etoposide in neuronal SHSY5Y cells (Teng and Tang, 2013).

RTN3A1 is a potent modulator of Alzheimer's disease pathogenesis. Beta-site amyloid precursor protein-cleaving enzyme 1 (BACE1) promotes processing of amyloid precursor protein (APP) into beta-amyloid (Aβ), which can form senile plaques in the brain (Masters *et al.*, 2015). Inhibition of BACE1 is a putative therapy for Alzheimer's disease, and RTN3A1 indirectly inhibits BACE1 activity by two mechanisms. First, *in vitro* studies in hippocampal/cortical neurons show that RTN3A1 inhibits axonal transport of BACE1 into the synapse, which limits the interaction with APP, decreasing the levels of cleavage products (Deng *et al.*, 2013). RTN3A1 also decreases the total levels of BACE1 in the brain (Shi *et al.*, 2014). Consistent with these findings, Shi *et al.* (2009b) reported that Aβ plaque deposition in the cortex and in the hippocampus (CA3/DG) of 6-month-old APP/PS1 mice was decreased by overexpressing RTN3A1 in the brain. Multiple neurotoxic fragments (Aβ_1–40_ and Aβ_1–42_) were decreased in the cortex in RTN3A1 overexpressing mice (Shi *et al.*, 2009b). Similar results were reported by others (Araki *et al.*, 2013). Conversely, RTN3A1 gene KO in 6-month-old AAP/PS1 mice increased the rate of APP processing, and increased Aβ plaque deposition in the forebrain (Shi *et al.*, 2014). Finally, Zou *et al.* (2018) identified dysfunctional RTN3A1 mutants in Alzhermier's patients caused by single-nucleotide polymorphisms; overexpression of RTN3A1 mutants *in vitro* led to either decreased BACE1 gene expression or increased (i.e., aberrant) axonal transport in neurons. Thus, wild-type RTN3A1 is neuroprotective in the setting of Alzheimer's disease by targeting Aβ burden in critical brain structures.

RTN3 is also neurotoxic. Hu *et al.* (2007) reported that RTN3A1 overexpression in the normal adult mouse brain induced the formation of “RTN3 immunoreactive dystrophic neurites” (RIDNS). RIDNS are caused by polymerization of RTN3A1 monomers into toxic high-molecular-weight aggregates, which lead to the accumulation of abnormal tubular ER in axons (Sharoar *et al.*, 2016). Interestingly, RTN3A1 aggregates are increased in cultured primary mouse neurons treated with 20 nM Aβ_1–42_, demonstrating a potentially vicious cycle between fibril plaque formation and RIDNS neuropathology (Hu *et al.*, 2007). Increased RIDNS burden in the mouse brain correlated with greater learning impairment on the Barnes maze, and disrupted electrophysiology in hippocampal slices *ex vivo* (Hu *et al.*, 2007). Furthermore, RIDNS develop naturally and are detected in the CA1 hippocampus in 24-month-old wild-type mice (Shi *et al.*, 2009a). Neuronal spine density is decreased in the CA1 in mice with increased RINDS burden. Finally, preventing the formation of RTN3A1 aggregates in CA1, using a conditional KO approach, increased hippocampal BDNF levels, and preserved contextual-cued memory on a fear conditioning paradigm (Shi *et al.*, 2013).

The complex interplay between the activation of RTN3-mediated neuroprotective versus neurotoxic mechanisms suggests the need to elucidate the consequences of increased RTN3 expression in the setting of TH. It is unclear if RTN3 activates different cell signaling programs depending on the temperature (e.g., 37°C vs. 33°C vs. 16°C) or age (e.g., adults vs. infants). Also, the benefits versus risks of increased RTN3 may differ depending on the type of acute brain insult. For instance, Zhao *et al.* (2013) reported that 300 μM H_2_O_2_ robustly increased high-molecular-weight (toxic) RTN3 aggregates in human neuronal SHSY5Ys. Oxidative stress is increased during a cardiac arrest, and the majority of oxidative brain damage occurs early after global ischemia (Wiklund *et al.*, 2018). Studies in piglets showed that TH to ∼33°C had limited efficacy to prevent oxidative brain injury after a cardiac arrest if cooling was initiated 30 minutes post-ROSC (Wiklund *et al.*, 2018). TH is generally applied within 6 hours post-ROSC clinically. Augmenting RTN3 in the setting of cardiac arrest may be detrimental because intracellular conditions already favor (before hypothermia) the formation of RTN3 aggregates. However, the development of IV therapies that immediately block oxidative brain damage before or early after reperfusion might prevent RTN3 aggregation and favor its neuroprotective effects. Another example is in the setting of a TBI, which is associated with increased Aβ plaques in the brain (Tran *et al.*, 2011) [see Johnson *et al.* (2010) for additional review]. Inhibition of BACE1 by TH-induced RTN3 may represent a feasible therapeutic strategy to reduce Aβ deposition after CNS trauma. In summary, RTN3 is one of the newest members of the CSP family. More research is needed to clarify its potential role(s) in the setting of acute brain injury, to determine if it is induced by clinically relevant levels of hypothermia *in vivo*, and if it represents a beneficial or detrimental component of the cold shock response.

## Hypothermia in a Syringe: An Auxiliary Approach to Cooling in the Neuro-ICU and a Case Example in Managing the Sickness of Long-Duration Spaceflight

A variety of approaches have been investigated to chemically induce hibernation-like physiology in humans. Safar *et al.* (2000) in the early 2000s reported remarkable brain tissue sparing effects of 60–120 minutes “suspended animation” (SA) also called EPR, by infusion of 2–4°C saline in exsanguinated dogs (Woods *et al.*, 1999; Behringer *et al.*, 2003). Woods *et al.* (2000) later tested if adenosine, a neuroactive compound that regulates hibernation in squirrels, augmented hypothermic neuroprotection with saline flush in that model (Jinka *et al.*, 2011). Hydrogen sulfide (H_2_S) is another promising agent to induce SA-like states (Blackstone *et al.*, 2005). However, Hemelrijk *et al.* (2018) recently contested its long-assumed mechanism of action by showing that H_2_S does not cause hypothermia in normoxic mice (FiO_2_ 0.21) but rather augments hypoxia-induced anaprexia at an FiO_2_ of 0.17 or 0.05. H_2_S also failed to induce hypothermia in large animals such as pigs or piglets (Li *et al.*, 2008; Drabek *et al.*, 2011).

**FIG. 6.**
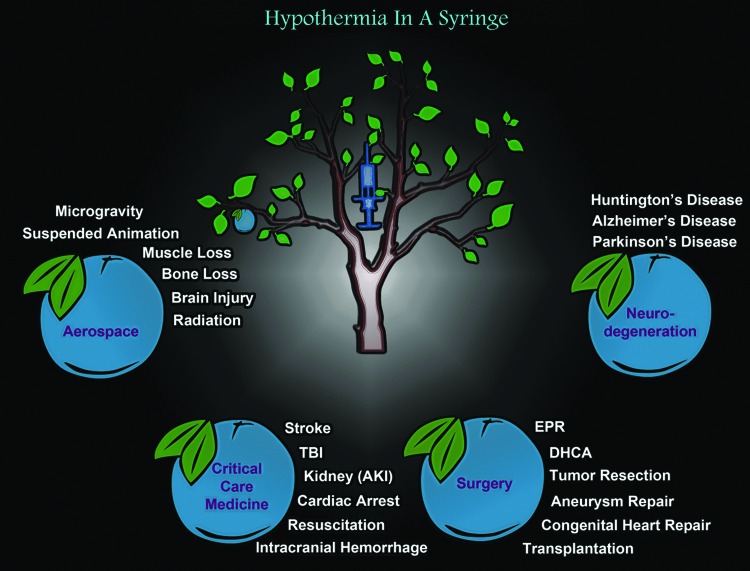
Medical conditions amenable to a “Hypothermia in a Syringe” strategy. Hypothermia in a Syringe is the theoretical concept that CSH/CSP levels can be (collectively) pharmacologically manipulated in normothermic or hypothermic patients to mimic (or augment) aspects of beneficial cold response physiology and cell signaling cascades. The illustration shows examples of major research fields (individual fruits), and medical conditions relevant to each (*white text*), which may represent low-hanging fruit for clinical translation. Prioritization of the “lowest hanging fruit” is left to the reader to interpret given that the metaphor is context dependent (e.g., the lowest hanging fruit might represent a condition most applicable to a therapy, or alternatively a condition via which a therapy is able to translate into the clinic the fastest).

How might “Hypothermia in a Syringe” differ from prior concepts to chemically induce neuroprotective cooling? A key difference is that cooling is not the main objective. Rather, the objective is to recreate the cell signaling environment activated by CSH/CSP responses during cold stress, but (optionally) with or without hypothermia. In this way Hypothermia in a Syringe could be applied to normothermic patients in whom complex cooling instrumentation is simply not practical (e.g., artic sun device in conscious Alzheimer's patients), or takes time to induce. Similarly, a Hypothermia in a Syringe approach, if accomplished at normothermia, could be implemented without any of the traditional side effects of cooling. Hypothermia in a Syringe might also be able to synergize with additional mechanisms of neuroprotection induced by TH in patients (Fig. 6). In theory, we envision a drug mixture that might comprise a combination of agents such as FGF21, Meternl, Irisin, SHBG, RBM3 agonist, CIRBP agonist, and/or an RTN3 agonist (and other components added or excluded). Notably, the concepts of “depth and duration” might also be relevant to Hypothermia in a Syringe. The magnitude increase (or decrease) of endogenous CSHs/CSPs varies based on the depth and duration of hypothermia in cells. Thus, target tissue levels achieved via an artificial approach (i.e., based on IV loading dose) might replicate unique states of altered cell signaling corresponding to specific hypothermic “depths,” and would need to be optimized. The concept of Hypothermia in a Syringe is certainly speculative, but arguably, it dovetails with additional ideas to improve the efficacy of TH in the treatment of acute or chronic brain injury. It might take decades for such a combinatorial therapy to be developed, and other adjuncts beyond simply drug administration may be necessary. Recognizing the speculative nature of this concept, we discuss its potential utility in one of the most logical and potentially useful setting, namely, exploration of deep space.

NASA's Human Research Program is expected to publish the integrated findings on the Twins Study (www.nasa.gov/feature/nasa-twins-study-investigators-to-release-integrated-paper-in-2018) in the coming months. This historic report represents the largest genomic analysis ever done to understand the long-term effects of space on the human body, and reinforces the extent that investments are being made to improve health care in astronauts. In addition, numerous stakeholders in the Aeronautics and Astronautics industry have proposed plans to travel to Mars by the late 2020–2030s, or to house tourists inside orbital or suborbital enclosures for extended excursions. For these efforts to succeed, medicines must be developed to combat the potentially harmful physiological changes induced by weightlessness, prevent tissue damage caused by increased exposure to radiation, and simplify the delivery of advanced medical techniques in microgravity. Kirkpatrick *et al.* (2009) speculated on the potential utility of chemically induced SA in astronauts in the setting of emergency surgical repair of traumatic injuries during exploration class missions. Similarly, Cerri *et al.* (2016) discussed the utility of adenosine precursor 5-AMP to induce hibernation-like torpor during long-distance space travel (Cerri *et al.*, 2016). How might Hypothermia in a Syringe synergize with these concepts?

**FIG. 7.**
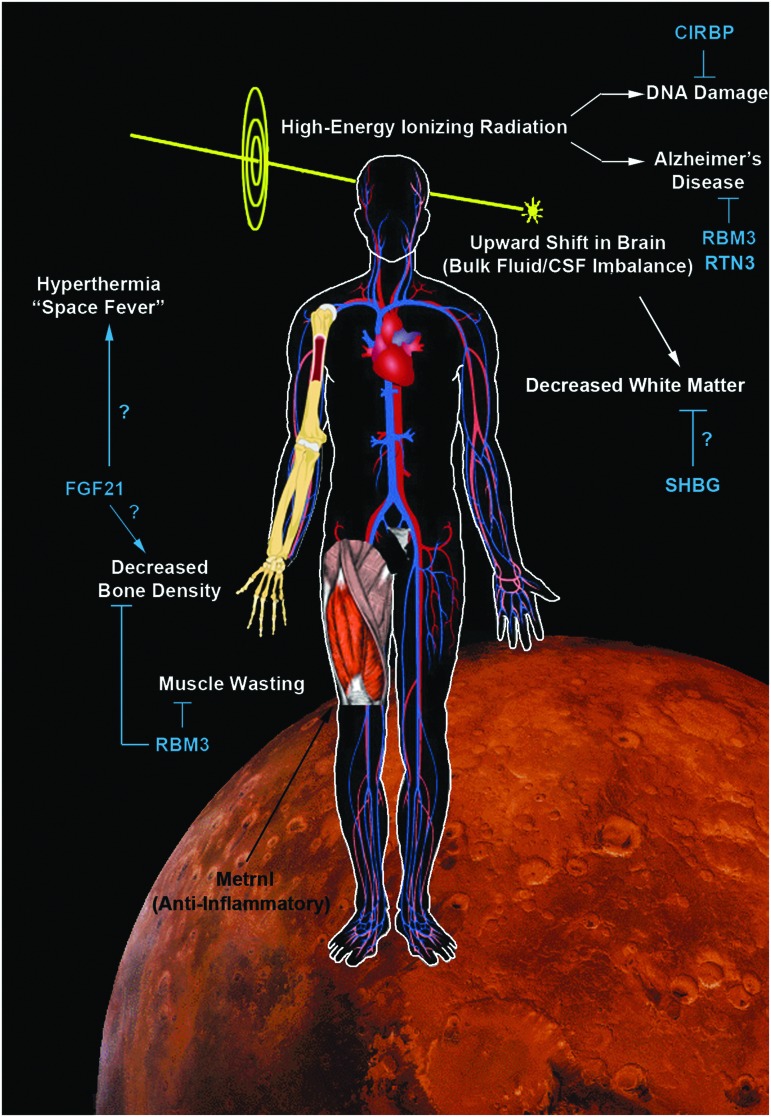
Theoretical multiorgan benefits of Hypothermia in a Syringe in the setting of long-duration spaceflight. Astronauts exposed chronically to microgravity and increased levels of radiation have evidence of cumulative tissue damage, and show marked changes in baseline physiology that may predispose organs such as the brain to a state of enhanced vulnerability. The utility of protective cooling in astronauts is a well-recognized potential therapy to prevent tissue damage during long-duration spaceflight. However, the technology involved in the implementation of cooling systems and protocols able to safely maintain a state of suspended animation in humans is incomplete. Moreover, adopting standard hypothermia equipment (e.g., the artic sun system) for a shuttle or medical module, and used for acute cooling in the event of an accidental injury (e.g., traumatic brain injury), will require overcoming design and training challenges to ensure patient safety in microgravity. Thus, in the relative near term, developing IV-based therapeutics that quickly and easily activate neuroprotective cold responsive signaling pathways in astronauts, and without the need for cooling, may represent a technically simpler approach for space travel (although not necessarily a replacement for the latter). Germane to that logic, key CSPs discussed in this review show promise in reversing physiological changes and cellular pathologies that appear to be associated with spaceflight. For instance, RBM3 (1) prevents cognitive impairment in models of Alzheimer's disease, (2) protects against muscle atrophy, and (3) may decrease bone loss. CIRBP promotes DNA repair mechanisms after ionizing radiation (but this effect is yet to be tested in neurons of the brain). Finally, RTN3 decreases Aβ plaque burden in the brain. CSHs may also mediate beneficial and/or detrimental effects on altered physiology in astronauts. In particular, Metrnl is associated with pathways involved in muscle strength and anti-inflammatory signaling in WAT. In addition, higher SHBG plasma levels are associated with increased white matter in men, but whether that relationship is causative or correlative remains unclear, and also needs to be investigated in women. Finally, FGF21 may activate neuroprotective pathways in the brain, or conversely, further promote a detrimental “space fever” phenotype in astronauts by activating heat-generating thermogenesis mechanisms in BAT. Aβ, beta-amyloid; IV, intravenous.

Multiple physiological changes or injury mechanisms induced by long-duration spaceflight are also modulated by CSH/CSP pathways (Fig. 7). Muscle atrophy is a well-known consequence of long-duration spaceflight (Fitts *et al.*, 2010). Overexpression of RBM3 in myoblasts inhibits cell death induced by H_2_O_2_ (necrosis) or staurosporine (apoptosis) (Ferry *et al.*, 2011). Van Pelt *et al.* (2018) showed that RBM3 overexpression in the soleus muscle in rats increased muscle fiber size and decreased atrophy after 14 days of hind limb suspension. Furthermore, Metrnl is activated by upstream signaling pathways involved in muscle hypertrophy/strength, is secreted by the muscles during exercise, and increases the release of anti-inflammatory molecules (Rao *et al.*, 2014).

Bone loss is another complication of space travel (Smith *et al.*, 1999). RBM3 overexpression in MC3T3-E1 osteoblasts increased mRNA and protein levels of Runx2 (a TF that stimulates osteoblast differentiation) (Kim *et al.*, 2018). However, it is unclear if some CSH/CSP pathways may be detrimental in microgravity. Transgenic mice overexpressing FGF21 (fivefold higher levels vs. wild type) had decreased bone mass (Wei *et al.*, 2012). However, this finding was not replicated by others, and bone homeostasis remained normal in mice administered 3 mg/kg rhFGF21 for 2 weeks (Li *et al.*, 2017c). Also, bone mass was not increased in FGF21 KO mice (Li *et al.*, 2017c).

Radiation from galactic cosmic rays (GCR) and solar energetic particles is a major health concern for astronauts. Zeitlin *et al.* (2013) analyzed data collected from the Mars Science Laboratory Spacecraft during its 253-day mission to Mars, and estimated that humans making the same journey would have an accumulative radiation exposure equivalent to ∼42–50 total body computed tomography scans (i.e., based on the standard exterior shielding technology used on spacecraft at the time of the study). Furthermore, Cherry *et al.* (2012) showed that clinically relevant GCR exposure levels (i.e., germane to astronauts) in 7–9.5-month-old APP/PS1 mice increased Aβ deposition in the brain, and exacerbated learning and memory impairments versus nonradiated controls. RBM3 overexpression in the brain decreased Alzheimer's disease neuropathology and cognitive impairment in 5XFAD mice (Peretti *et al.*, 2015). Also, RTN3 overexpression in the brain decreased Aβ deposition.^70,173^ Thus, RBM3 and RTN3 may help to defend the brain against the development of Alzheimer's disease pathology during long missions. Furthermore, Chen *et al.* (2018c) demonstrated that CIRBP is an essential component of the molecular machinery that mediates DNA repair after ionizing radiation. Knockdown of CIRBP in U2OS cells inhibited double-strand break (DSB) repair by ∼50%; both homologous recombination and nonhomologous end-joining mechanisms were negatively affected. We reported that CIRBP is absent in the hippocampus and in the cortex in adults (Jackson *et al.*, 2018). Thus, CIRBP inducing drugs may augment DNA repair mechanisms in the brain (although more studies are needed to confirm that CIRBP regulates DSB repair in neurons).

Microgravity also causes (1) Spaceflight-Associated Neuro-ocular Syndrome (SANS), with accompanying increased ICP, and (2) emergent hyperthermia (“space fever”) (Stahn *et al.*, 2017; Zhang and Hargens, 2018). MRI of the brain in astronauts returning from long-duration missions revealed a surprising upward shift in the brain/brainstem, which compressed structures at the vertex, including the central sulcus (Roberts *et al.*, 2017). This phenomenon may constrict venous/CSF drainage via the superior sagittal sinus and contribute to increased ICP. Also, CSF volume is increased in the subarachnoid space 7 months after returning to the Earth (Van Ombergen *et al.*, 2018). Furthermore, MRI revealed a persistent decrease in cerebral white matter volume after long-duration missions (Van Ombergen *et al.*, 2018). Prophylactically increasing neuroprotective CSPs may increase the threshold for secondary brain insults in the event of an accidental TBI. Studies are needed to test if SHBG might target white matter loss (Elbejjani *et al.*, 2017). Interestingly, *in vitro* studies showed that simulated microgravity decreased SHBG levels in cultured Sertoli cells (Masini *et al.*, 2011). However, SHBG blood levels in astronauts did not significantly change after 180 days on the international space station (Smith *et al.*, 2012).

Resting body temperature gradually increases in microgravity and plateaus to ∼37.5–38°C by 75 days in spaceflight (Stahn *et al.*, 2017). Furthermore, T_b_ increases even further (∼40°C) during strenuous excise (Stahn *et al.*, 2017). This so-called space fever may result from a decreased capacity for convection/evaporative heat loss, and also by increased stimulation of heat generating mechanisms (e.g., chronic inflammation) (Sonnenfeld, 1994; Stahn *et al.*, 2017). Small increases in T_b_ exacerbate brain injury and decrease the levels of both RBM3 and CIRBP (Nishiyama *et al.*, 1997; Sakurai *et al.*, 2012; Wong *et al.*, 2016). Thus, endogenous neuroprotective CSPs may be decreased in astronauts. Also, persistent hyperthermia might decrease the efficacy of CSP inducing drugs. Finally, it is unclear if CSHs (FGF21, Metrnl, or Irisin) might exacerbate hyperthermia in astronauts, by increasing the activation of nonshivering thermogenesis mechanisms, and thus impede their potential CNS benefits. The futuristic concept of Hypothermia in a Syringe may have applications in space travel. However, research is needed to define which cold stress targets offer the greatest therapeutic benefits with the lowest potential side effects.

## Concluding Remarks

Hypothermia remains an important clinical tool for improving patient outcomes across a spectrum of medical conditions. In addition, significant technological and conceptual advances in the delivery of health care over the last century have dramatically decreased mortality in the hospital setting, and increased the life span at the population level. These remarkable societal achievements have raised the bar to detect a benefit of cooling in CNS injury when superimposed on the many other global improvements in medicine that represent the dynamism of modern background care. This has perhaps, in part, led to the dawn of the TTM era in critical care, and a growing recognition that preventing fever may be one of the most important effects mediated by TH (excluding infants with HIE) in patients in modern ICUs—an effect that can be achieved at temperatures well above levels classically defined as neuroprotective. At the same time, results of animal studies are undeniable—traditional levels of TH in young or adult rodents are remarkably protective during or after a brain injury and these effects are highly reproducible across time, disease models, and species. Whether or not TH can be further optimized in humans across the age spectrum is an open question. Many strategies to enhance neuroprotective cooling have been proposed and all of these deserve further exploration.

In this review, as stated in the Introduction, we have tried to accomplish several key goals. First, to link emerging evidence in endocrinology, hibernation, neurocritical care, and brain injury research, often viewed in isolation, but together suggesting novel approaches that may lead to unique understanding and utility of TH. Second, to focus the review on the heretofore understudied novel neuroprotective mechanisms activated by TH, in preclinical and clinical models of acute CNS injury, and discuss how they may hold potential clues for improving the efficacy of TH in the treatment of acute and chronic CNS injury in humans, and beyond. These mechanisms, we believe hold promise for unique potential applications, including use and optimization of UMH, modulation of the endogenous Responsivity of Cold Stress Pathways, and finally, even potential development and application of the futuristic concept of Hypothermia in a Syringe. These novel concepts merit further investigation.
